# Restrictive Versus Liberal Fluid Strategy for Initial Resuscitation in Sepsis and Septic Shock: A Systematic Review and Meta Analysis

**DOI:** 10.14740/jocmr6464

**Published:** 2026-03-26

**Authors:** Ahmed Osman Ali, Hossam Ahmad Adib Kordi, Marwa Hussein Said Ahmad Alhag, Abdelghafar Mohamed Abdelghafar Salama, Zahra Gumaid Alqarni, Abdallah Dwayat, Alam Eldin Musa Mustafa, Abdelhadi Okasha, Shouq Abdullah, Nada Saleem Mohammed Alali, Zainab Saleem Mohammed Alali, Manal SA Hakami, Yazan Yousef A. Alghammas, Niemat Mohammed Tahir Ali, Ali Alghannami

**Affiliations:** a Department of Critical Care, Dr. Soliman Fakeeh Hospital, Riyadh, Saudi Arabia; b Emergency Department, Abha International Private Hospital, Abha, Saudi Arabia; c Department of Pediatric Emergency, Maternity and Children Hospital, Hail, Saudi Arabia; d Department of Emergency Medicine, Alghad College for Applied Medical Sciences, Riyadh, Saudi Arabia; e Intensive Care Unit, King Fahad Hospital, Madinah, Saudi Arabia; f Faculty of Medicine, Al-Quds University, Jerusalem, Palestine; g Department of Child Health, College of Medicine, King Khalid University, Abha, Saudi Arabia; h Department of Pediatrics, Faculty of Medicine and Health Sciences, University of Kordofan, El Obeid, Sudan; i Jordan University Hospital, Amman, Jordan; j Prince Sultan Military College of Health Sciences (PSMCHS), Al Amal, Dhahran, Saudi Arabia; k Manchester Program for Medical Education, Mansoura, Egypt; l Research Science in Nursing and Midwifery, Department of Material and Child Health Nursing, Faculty of Nursing, Al-Baha University, Al-Baha, Saudi Arabia; m College of Surgeons in Ireland, Dublin, Ireland; n Ministry of Health, Riyadh, Riyadh Region, Saudi Arabia

**Keywords:** Trial sequential analysis, Acute kidney injury, Restrictive fluid, Sepsis

## Abstract

**Background:**

Intravenous fluid resuscitation is essential in early management of sepsis, but the optimal volume and resuscitation strategy are uncertain. This systematic review and meta-analysis aimed to synthesize the evidence comparing the efficacy and safety of restrictive versus liberal fluid resuscitation strategies in adults with sepsis or septic shock.

**Methods:**

A systematic search of PubMed, Web of Science (WoS), Scopus, and CENTRAL was conducted from inception to November 2025, following the Preferred Reporting Items for Systematic Reviews and Meta-Analyses (PRISMA) and Meta-analysis of Observational Studies in Epidemiology (MOOSE) guidelines. Randomized controlled trials (RCTs) and observational cohort studies comparing protocolized restrictive fluid strategies with liberal or standard care were included. Primary outcomes were all-cause mortality and acute kidney injury (AKI). Random-effects models were used to calculate pooled risk ratios (RRs) and mean differences (MDs), while trial sequential analysis (TSA) assessed the conclusiveness of evidence.

**Results:**

Sixteen reports from 15 unique studies (nine RCTs and six observational studies) involving 5,013 patients were included. In the analysis of RCTs, restrictive fluid therapy resulted in no significant difference in all-cause mortality (RR = 0.99; 95% CI, 0.90–1.08; I^2^ = 0%); however, observational studies showed a significant mortality reduction (RR = 0.69), suggesting confounding by indication in observational datasets. TSA of the RCT data indicated insufficient evidence for mortality benefit. Restrictive strategies were associated with a lower risk of AKI (RR = 0.89; 95% CI, 0.81–0.99; P = 0.02; I^2^ = 0%) and a reduced incidence of acute respiratory distress syndrome (ARDS) (RR = 0.69; 95% CI, 0.56–0.85; P < 0.001; I^2^ = 0%). No significant differences were observed in other ischemic, metabolic, or organ support outcomes. However, restrictive fluid therapy reduced the need for mechanical ventilation, shortened the ventilation duration and vasopressin use, and increased ventilator-free days.

**Conclusions:**

Restrictive fluid resuscitation does not reduce overall mortality in adults with sepsis or septic shock, but it is associated with lower AKI and ARDS risk and decreased dependence on mechanical ventilation. Evidence regarding mortality is inconclusive, highlighting the need for large-scale trials to validate this finding.

## Introduction

Sepsis is a life-threatening organ dysfunction accompanied by hypotension because of peripheral vasodilation, myocardial depression, and fluid extravasation [1]. It is a major cause of morbidity and mortality, accounting for millions of deaths every year, highlighting the need for continuous improvements in clinical management and supportive care [2, 3]. Intravenous (IV) fluid resuscitation is an essential treatment for early onset sepsis, aimed at increasing depleted intravascular volume to restore tissue perfusion and improve circulation caused by the sepsis-induced vasodilated vascular network [4, 5]. Although early administration of IV fluids is critical for managing sepsis, the optimal fluid administration is debated [6].

The current Surviving Sepsis Campaign (SSC) guidelines recommend administering at least 30 mL/kg of IV isotonic crystalloid within the first 3 h for patients with sepsis-induced hypoperfusion or septic shock [6]. SSC recommends a fixed-volume fluid for initial resuscitation, but the evidence supporting this volume is of low certainty, and the guidelines offer no clear direction regarding whether a restrictive or liberal strategy should be used during the initial resuscitation phase [7]. According to the SSC fixed-dose recommendation, two-thirds of patients with septic shock exhibit fluid overload, dilutional coagulopathy, and pulmonary edema [8, 9].

Also, evidence from randomized trials in patients with sepsis and septic shock indicates that administering higher volumes of IV fluids may be harmful, with reported adverse outcomes including worsening of kidney injury [10], respiratory failure [11], and a higher risk of death [12]. The optimal strategy remains unclear after the neutral findings of the CLASSIC [13] and CLOVERS trials [14] regarding whether a restrictive fluid strategy is associated with a lower incidence of all-cause mortality compared to the control group in patients presenting with sepsis or septic shock.

A critical limitation in interpreting current evidence is the variation in “initial” resuscitation; notably, major trials such as CLASSIC and CLOVERS randomized patients only after they had already received substantial pre-enrolment fluid boluses (often > 1–2 L), effectively testing a “post-initial-bolus” restrictive strategy rather than a “door-to-needle” restrictive strategy.

Because of the conflicting results in the literature and the lack of evidence supporting SSC recommendations regarding the optimal fluid administration strategy during the initial resuscitation phase, this systematic review and meta-analysis was conducted to evaluate all-cause mortality and fluid-related adverse outcomes in adult patients with sepsis or septic shock.

## Materials and Methods

This systematic review and meta-analysis was designed and conducted following the Preferred Reporting Items for Systematic Reviews and Meta-Analyses (PRISMA) guidelines and the methodological standards outlined in the Cochrane Handbook for Systematic Reviews of Interventions and Meta-analysis of Observational Studies in Epidemiology (MOOSE) recommendations [14–16]. The protocol was registered in PROSPERO under registration number (CRD420251182239). As this study is a systematic review and meta-analysis of previously published data, ethical approval and patient consent were not required.

### Data sources and search strategy

The studies included in this meta-analysis were retrieved through a search across electronic databases, including PubMed (MEDLINE), Web of Science (WoS), Scopus, and the Cochrane Central Register of Controlled Trials (CENTRAL) from inception to November 1, 2025. The search strategy was constructed using Boolean operators, incorporating the following search terms: ((“sepsis” OR “septic shock”) AND (“restrictive fluid” OR “fluid sparing” OR “liberal” OR “usual care” OR “standard care”)). The full search strategy for each database is detailed here (Supplementary Material 1, jocmr.elmerjournals.com). Manual backward and forward citation tracking were performed to enhance the completeness of the search as backward citation analysis involved reviewing the reference lists of included studies and relevant reviews, while forward citation analysis used platforms such as Google Scholar and Scopus to identify newer studies that cited the included articles.

### Study selection

All the records retrieved from the search were imported into EndNote to remove duplicates, while the remaining citations were uploaded to Rayyan for screening [17]. Study selection was conducted in two stages based on eligibility criteria. In the first stage, two independent reviewers screened titles and abstracts to identify potentially eligible studies. In the second stage, the full texts of these studies were assessed in detail to determine the inclusion of randomized controlled trials (RCTs) and observational studies that met the prespecified PICO framework. Disagreements were resolved through discussion, and a third reviewer adjudicated any unresolved conflicts to ensure methodological rigor.

### Eligibility criteria and outcomes

RCTs and observational cohort studies enrolling adult patients (≥ 18 years) with sepsis or septic shock based on contemporary international definitions and managed during the initial resuscitation phase, defined as the first 24 h after the recognition of sepsis-induced hypoperfusion were included. For many included RCTs, this intervention period began after an initial standard-of-care fluid bolus had already been administered prior to randomization. The intervention of interest was a protocolized restrictive fluid resuscitation strategy involving deliberate limitation of cumulative fluids, early vasopressor initiation, or predefined physiological triggers that guide additional fluid use. Eligible comparators were liberal, or standard-care fluid strategies characterized by higher-volume fluid administration, typically ≥ 30 mL/kg, and conventional fluid-challenge-based hemodynamic assessment within the same 24-h period. We excluded studies involving children or adolescents aged < 18 years, cardiogenic or hemorrhagic shock, late (> 24 h) fluid intervention following initial resuscitation, non-protocolized or poorly defined strategies, fluid-type comparisons without volume differences, studies without comparable control groups, and case reports, case series, conference abstracts, and non-adjusted observational studies.

The primary outcomes included both efficacy and safety. The primary efficacy outcome was all-cause mortality, whereas the primary safety outcome was the incidence of acute kidney injury (AKI), which was assessed at the longest available follow-up. The secondary clinical outcomes included ischemic events (cerebral, intestinal, limb, or myocardial ischemia), disseminated intravascular coagulation (DIC), multiple organ dysfunction syndrome (MODS), intensive care unit (ICU) admission rate, pulmonary edema, and the overall incidence of serious adverse events. Electrolyte and metabolic outcomes, including hyperchloremia, hypernatremia, hyponatremia, and hypoglycemia, were also evaluated. Additional efficacy outcomes included the need for mechanical ventilation, vasopressin requirement, initiation of renal replacement therapy (RRT), hospital and ICU length of stay, duration of mechanical ventilation and vasopressin use, days free from organ support, vasopressor-free days, and ventilator-free days.

### Data extraction and risk of bias assessment

Data extraction was performed independently and duplicated by four authors using a standardized Microsoft Excel form as the extracted variables included the study characteristics, patient demographics, and all reported outcomes, while all entries were cross verified to ensure accuracy, and discrepancies were resolved by consensus.

The methodological quality of the included studies was evaluated using the Cochrane Risk of Bias-2 (RoB-2) tool for RCTs [18] and the Newcastle–Ottawa Scale (NOS) [19] for observational studies. The RoB-2 tool examined six domains: random sequence generation, allocation concealment, blinding of participants and personnel, blinding of outcome assessors, completeness of outcome data, and selective reporting. Each domain was classified as having a low risk, some concerns, or high risk of bias. For observational studies, the NOS assigns up to 9 points across three domains: selection (4 points), comparability (2 points), and outcome assessment (3 points) as overall NOS scores of 0–3 denote poor quality, 4–6 moderate quality, and 7–9 good quality, respectively. Any discrepancies between reviewers were resolved through discussion.

### Statistical analysis and heterogeneity assessment

All statistical analyses were performed using STATA 17 MP and were conducted according to the intention-to-treat analysis, whereby all randomized participants were analyzed in their originally assigned groups. A random-effects model based on the Der Simonian–Laird method was used to estimate risk ratios (RRs) for dichotomous outcomes and mean differences (MDs) for continuous outcomes. Stratified analyses were performed separating RCTs from observational studies for all primary outcomes to account for significant methodological heterogeneity and the risk of confounding by indication. Overall pooled estimate combining both study designs was not calculated, as this would obscure the higher certainty evidence provided by RCTs. For studies reporting outcomes at multiple long-term follow-up points, the latest available time points were used for meta-analysis. Statistical heterogeneity was assessed using the Chi-square test and quantified using the I^2^ statistic, I^2^ = ((Q–df)/Q) × 100%. A P value < 0.1 was considered indicative of significant heterogeneity, and I^2^ values ≥ 50% were interpreted as reflecting substantial heterogeneity. For outcomes where I^2^ = 0%, we carefully reviewed the forest plots to distinguish between true consistency of effect versus sparse data or wide overlapping confidence intervals (CIs). In cases of significant heterogeneity, a leave-one-out sensitivity analysis was conducted to evaluate the robustness of the findings and determine the influence of individual studies on the pooled estimates. Galbraith plots were constructed to detect potential outlier studies that contributed to significant heterogeneity. L’Abbe plots were used to compare the event rates between the intervention and control groups, while funnel plots were generated to evaluate the relationship between the effect size and standard error, identifying inconsistencies and potential publication bias across the included studies.

Furthermore, a trial sequential analysis (TSA) [20] was conducted to compare the restrictive fluid strategy with the control group and reduce the risk of type I error associated with repeated significance testing in cumulative meta-analyses. The TSA parameters were defined as a two-sided α of 0.05, a β of 0.20 (80% power), and an anticipated relative risk reduction of 20%. The diversity-adjusted information size (DIS) was calculated using the heterogeneity (D^2^) observed in the meta-analysis. The control event rate was derived from the pooled incidence observed in the control arms, and the required information size (RIS) was calculated. Evidence was considered conclusive if the cumulative Z-curve crossed both the conventional significance boundaries and the trial sequential monitoring boundaries or if it reached the RIS. If these conditions were not met, the evidence was inconclusive, indicating the need for additional RCTs.

Several subgroup analyses were conducted to explore the potential sources of between-study heterogeneity. For all-cause mortality, subgroup analyses were stratified by study design (RCTs vs. observational studies), and the time point at which mortality was assessed (in-hospital, 28-day, 30-day, 60-day, or 90-day). Additionally, subgroup analysis was performed for AKI stratified according to AKI stage.

## Results

### Literature search

**Figure 1:**
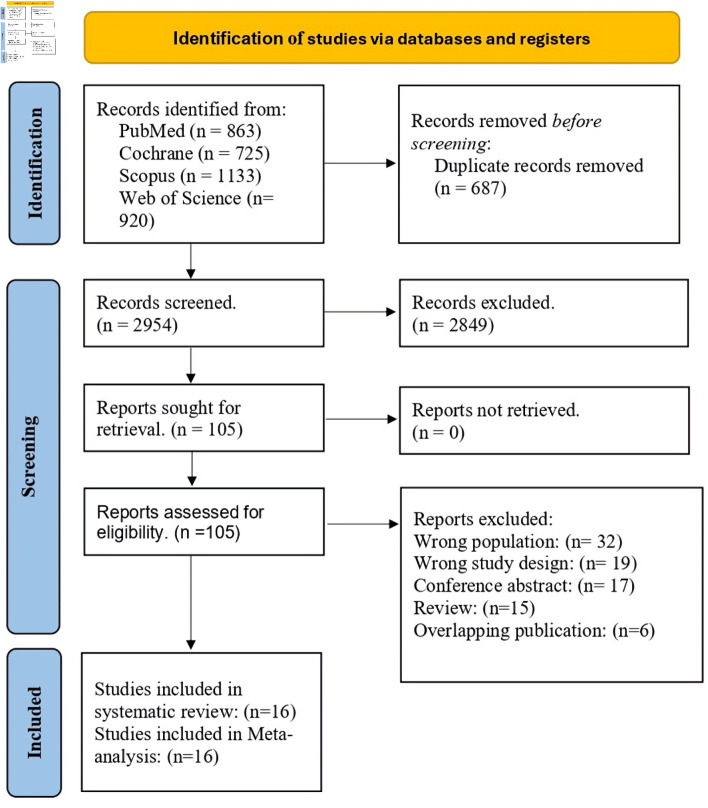
PRISMA flow diagram. PRISMA: Preferred Reporting Items for Systematic Reviews and Meta-Analyses.

The literature search yielded 3,641 records. After removing 687 duplicate studies, 2,954 remained for title and abstract screening. Of these, 2,849 were excluded based on title and abstract reviews, leaving 105 full-text articles assessed for eligibility. Following full-text screening, 16 reports representing 15 unique studies met the predefined inclusion criteria and were included in the quantitative synthesis [13, 14, 21–34]. The study selection process is illustrated in the PRISMA flow diagram in Figure 1.

### Characteristics and risk of bias assessment of the included studies

Sixteen reports from 15 unique studies were included in this meta-analysis, consisting of nine RCTs and six observational cohort studies. Across all studies, the pooled mean age of participants was 63.4 years, and 3,656 (72.9%) were male. A restrictive fluid strategy was administered to 2,584 (51.5%) patients, while 2,429 (48.5%) patients received a liberal fluid regimen, serving as the control group among individuals presenting with sepsis or septic shock. Follow-up durations varied across the studies, ranging from 28 to 365 days, with a mean follow-up duration of 75.9 days. A detailed summary and baseline characteristics of each included study are presented in Tables 1 and 2 [13, 14, 21–34]. Quality assessment using RoB-2 for RCTs indicated a low risk of bias for all studies, except for the study by Douglas et al (2020) [34] and Macdonald et al (2018) [26], and the NOS for observational studies demonstrated a high overall quality rating (Supplementary Materials 2, 3, jocmr.elmerjournals.com).

**Table 1:** Baseline Characteristics of the Included Patients

Study ID	Groups	Patient demographics			Hemodynamic parameters, mean (SD)			Focus of infection, n (%)			Comorbidities, n (%)		
		Age (years), mean (SD)	Weight (kg), mean (SD)	Male, n (%)	SOFA score	Lactate, mmol/L	Creatinine (µmol/L)	Pulmonary	Urinary tract	Skin or soft tissue	Diabetes	Hypertension	CKD
Andrews et al, 2017 [33]	Restrictive fluid	35.8 (11.9)	NA	55 (53.4)	NA	4.5 (3.3)	141.5 (119.3)	NA	2 (1.9)	NA	NA	NA	NA
	Standard fluid	37.5 (12.9)	NA	62 (58.5)	NA	5.4 (4.4)	141.5 (88.4)	NA	2 (1.9)	NA	NA	NA	NA
Boulet et al, 2024 [21]	Restrictive fluid	69.5 (8.8)	67.7 (14.8)	12 (50)	NA	2.4 (1.8)	98.5 (52)	12 (50)	1 (4.2)	1 (4.2)	8 (33.3)	14 (58.3)	1 (4.2)
	Standard fluid	67.0 (11.5)	73.4 (16.6)	13 (54.2)	NA	2.2 (1.1)	158.8 (47.6)	11 (45.8)	5 (16.7)	1 (4.2)	5 (20.8)	11 (45.8)	2 (8.3)
Meyhoff et al, 2022 [13]	Restrictive fluid	70 (11.4)	78 (17)	452 (59.9)	NA	4.1 (2.4)	150.2 (84.8)	209 (27.7)	119 (15.8)	62 (8.2)	NA	346 (45.8)	9 (1.2)
	Standard fluid	69 (12.6)	78.6 (17.8)	452 (58.2)	NA	4.2 (2.4)	153.8 (91)	206 (26.5)	133 (17.1)	64 (8.2)	NA	360 (46.4)	12 (1.5)
Shapiro et al, 2023 [14]	Restrictive fluid	59.1 (16)	NA	411 (52.6)	3.4 (2.8)	2.9 (2.5)	159.12 (50.2)	217 (27.7)	148 (18.9)	97 (12.4)	222 (28.6)	NA	33 (4.2)
	Standard fluid	59.9 (15.9)	NA	415 (53.1)	3.5 (2.7)	2.9±2.4	167.9 (167.9)	205 (26.2)	172 (22.0)	82 (10.5)	224 (29.0)	NA	40 (5.2)
Corl et al, 2019 [22]	Restrictive fluid	71 (16.7)	87.7 (21.3)	24 (43.6)	8.7 (3)	2.3 (1.7)	NA	13 (23.6)	15 (27.3)	8 (14.6)	23 (41.8)	NA	7 (12.7)
	Standard fluid	69.5 (20.5)	80.1 (17)	26 (48.2)	9.1 (3.8)	3.2 (2.8)	NA	17 (31.5)	14 (25.9)	2 (3.7)	18 (33.3)	NA	1 (1.9)
Douglas et al, 2020 [34]	Restrictive fluid	61.8 (16.9)	73.7 (18.7)	32 (38.6)	NA	3.6 (3.2)	NA	NA	NA	NA	29 (34.9)	42 (50.6)	NA
	Standard fluid	62.7 (15)	73.6 (18.5)	28 (68.3)	NA	3.8 (3.6)	NA	NA	NA	NA	11 (26.8)	25 (61)	NA
Jessen et al, 2022 [23]	Restrictive fluid	75.7 (13.7)	77 (21.2)	37 (61)	2.7 (0.75)	1.4 (0.6)	98 (53.9)	45 (74)	9 (15)	3 (5)	11 (18)	NA	5 (8)
	Standard fluid	75.7 (11.3)	78.7 (15.9)	34 (55)	2.7 (0.75)	1.5 (0.8)	96 (50.8)	43 (69)	12 (19)	1 (2)	9 (15)	NA	9 (15)
Kjaer et al, 2023 [24]	Restrictive fluid	70 (11.4)	78 (17)	452 (59.9)	NA	4.1 (2.4)	150.2 (84.8)	209 (27.7)	119 (15.8)	62 (8.2)	NA	346 (45.8)	9 (1.2)
	Standard fluid	69 (12.6)	78.6 (17.8)	452 (58.2)	NA	4.2 (2.4)	153.8 (91)	206 (26.5)	133 (17.1)	64 (8.2)	NA	360 (46.4)	12 (1.5)
Linden et al, 2024 [25]	Restrictive fluid	71.4 (16)	82.7 (20.7)	26 (57)	10.4 (3.8)	4.2 (3.1)	172.4 (101)	13 (28)	12 (26)	5 (11)	NA	NA	NA
	Standard fluid	68.7 (13.7)	78.7 (22.1)	30 (61)	9.4 (3.8)	4 (2.7)	172 (148.16)	17 (35)	7 (14)	3 (6)	NA	NA	NA
Macdonald et al, 2018 [26]	Restrictive fluid	65.4 (19.8)	78 (16.7)	31 (62)	5.7 (4.5)	2.1 (1.8)	113.7 (64.8)	14 (28)	16 (32)	6 (12)	NA	NA	NA
	Standard fluid	62.4 (23.6)	75.4 (19.8)	30 (61)	5.4 (2.2)	1.8 (1)	126.7 (68.7)	20 (41)	9 (18)	6 (12)	NA	NA	NA
Hu et al, 2023 [27]	Restrictive fluid	66.28 (16.66)	NA	67 (59.82)	6.02 (2.97)	NA	147.29 (185.58)	NA	NA	NA	NA	NA	NA
	Standard fluid	67.25 (19.41)	NA	77 (60.63)	7.12 (2.99)	NA	94.03 (65.66)	NA	NA	NA	NA	NA	NA
Jiang et al, 2023 [28]	Restrictive fluid	64.8 (12.7)	NA	96 (68.1)	8.5 (3.4)	3.1 (2.1)	NA	53 (37.6)	18 (12.8)	8 (5.7)	29 (20.6)	57 (40.4)	NA
	Standard fluid	64.7 (13.8)	NA	61 (62.9)	9.3 (3.2)	3.9 (2.7)	NA	31 (32.0)	5 (5.2)	5 (5.2)	30 (30.9)	43 (44.3)	NA
Li et al, 2025 [29]	Restrictive fluid	56.24 (9.79)	NA	24 (58.5)	NA	NA	NA	16 (39)	NA	NA	8 (19.5)	19 (46.3)	2 (4.9)
	Standard fluid	56.56 (12.07)	NA	26 (63.4)	NA	NA	NA	15 (36.6)	NA	NA	NA	22 (53.7)	3 (7.3)
Rice et al, 2020 [30]	Restrictive fluid	71.2 (11.9)	88.3 (27.1)	28 (54.9)	7 (4)	NA	NA	NA	NA	NA	NA	NA	NA
	Standard fluid	72.5 (10.6)	84.9 (23.1)	27 (49.1)	6 (4)	NA	NA	NA	NA	NA	NA	NA	NA
Zhou et al, 2021 [32]	Restrictive fluid	40.19 (3.22)	60.19 (2.39)	30 (53.6)	NA	NA	NA	20 (35.7)	NA	14 (25)	NA	NA	NA
	Standard fluid	39.98 (3.35)	60.28 (2.31)	40 (60.6)	NA	NA	NA	23 (34.8)	NA	13 (19.7)	NA	NA	NA
Wang et al, 2021 [31]	Restrictive fluid	68.4 (15)	NA	78 (37)	12.3 (2.9)	4.8 (2.7)	NA	141 (67.4)	7 (3.3)	NA	36 (17.3)	48 (22.9)	8 (3.8)
	Standard-fluid	69.6 (13)	NA	28 (30)	12.3 (3.7)	4.9 (2.5)	NA	58 (62.4)	4 (4.3)	NA	17 (18.3)	33 (35.5)	3 (3.2)

Data are presented as: n (%) or mean (SD). NA: not available; SOFA: Sequential Organ Failure Assessment; CKD: chronic kidney disease; SD: standard deviation.

**Table 2:** Summary of the Included Studies

Study	Study design	Country	Time frame	Follow-up (days)	Sample size			Inclusion criteria	Primary outcomes
					Total	Restrictive strategy	Standard care		
Andrews et al, 2017 [33]	Parallel-group, non-blinded, randomized clinical trial (RCT)	Zambia	October 22, 2012, to November 11, 2013	28 days	209	103	106	Adults ≥ 18 years with sepsis (suspected infection + ≥ 2 SIRS criteria) and hypotension (SBP ≤ 90 mm Hg or MAP ≤ 65 mm Hg), enrolled within 4 h of the first eligible BP and within 24 h of ED registration	In-hospital mortality
Boulet et al, 2024 [21]	Single-blind, randomized, parallel, controlled pilot trial	France	September 20, 2021, to February 25, 2023	28 days	50	24	26	Adults 18–85 years, admitted to ICU with septic shock and within the first 24 h of vasopressor infusion	Cumulative fluid balance (mL/kg of admission body weight) in the first 5 days
Meyhoff et al, 2022 [13]	Open label randomized clinical trial	31 ICUs across Denmark, Norway, Sweden, Switzerland, Italy, Czech Republic, UK, and Belgium	November 27, 2018, to November 16, 2021	90 days	1,545	764	781	Adults ≥ 18 y in ICU with septic shock, received ≥ 1 L IV fluid in prior 24 h, onset of shock ≤ 12 h before screening	Death from any cause within 90 days
Shapiro et al, 2023 [14]	Randomized, unblinded superiority trial	USA	March 7, 2018, to January 31, 2022	90 days	1,563	782	781	Adults ≥ 18 years with suspected or confirmed infection who had sepsis-induced hypotension, defined in the trial as systolic blood pressure < 100 mm Hg after administration of ≥ 1,000 mL IV fluid	Death from any cause before discharge home by day 90
Corl et al, 2019 [22]	Prospective, randomized controlled trial	USA	November 2016 to February 2018	60 days	109	55	54	Adults admitted from the ED to the ICU with suspected severe sepsis or septic shock. After receiving a 1-L fluid bolus, they were included if they had persistent hypotension (MAP < 65 mm Hg) or lactate ≥ 4 mmol/L.	30-day all-cause mortality
Douglas et al, 2020 [34]	Prospective, multicenter, randomized, unblinded clinical trial	USA and UK	October 2016 to February 2019	30 days	124	83	41	Adults presenting to ED with sepsis or septic shock (paper used SIRS-based sepsis criteria: ≥ 2 SIRS criteria + suspected/documented infection) and anticipated ICU admission; refractory hypotension defined as MAP ≤ 65 mm Hg after receiving ≥ 1 L and < 3 L of fluid; enrolment within 24 h of hospital arrival	Difference in positive fluid balance at 72 h or ICU discharge
Jessen et al, 2022 [23]	Randomized, parallel-group, open-label feasibility trial	Denmark	November 3, 2021, to December 18, 2021	90 days	123	61	62	Adult age ≥ 18 years; sepsis defined by suspected infection by treating clinician, blood cultures drawn, IV antibiotics given or planned, infection-related increase in SOFA ≥ 2; expected hospital stay > 24 h	Total volume of IV crystalloid fluids administered during the first 24 h
Kjaer et al, 2023 [24]	Long-term follow of the CLASSIC trial	31 ICUs across Denmark, Norway, Sweden, Switzerland, Italy, Czech Republic, UK and Belgium	November 27, 2018, to November 16, 2021	365 days	1,549	767	782	Adults ≥18 years in ICU with septic shock, received ≥ 1 L IV fluid in prior 24 h, onset of shock ≤ 12 h before screening	Death from any cause within 1 year
Linden et al, 2024 [25]	Multicenter, parallel group, randomized feasibility trial	Sweden	March 7 and September 13, 2022	90 days	98	49	49	Adult patients (≥ 18 years of age) with septic shock (suspected/confirmed infection, plasma lactate > 2 mmol/L, and infusion of vasopressor to maintain MAP > 65 mm Hg after adequate fluid resuscitation) within 12 h of admission to the ICU and ongoing vasopressor therapy at the time of inclusion were eligible for inclusion	Total volume of fluid administered within 3 days
Macdonald et al, 2018 [26]	Multicenter, prospective, randomized, open-label clinical trial	Australia	October 2016 to March 2018	90 days	99	50	49	Adults with suspected infection requiring IV antibiotics and hypotension, defined as SBP < 100 mm Hg despite at least 1,000 mL isotonic crystalloid given within ≤ 1 h before randomization (SBP threshold was originally < 90 mm Hg, but changed to < 100 mm Hg early due to slow recruitment	Cumulative total IV fluid volume administered at 6 h post-randomization
Hu et al, 2023 [27]	Retrospective cohort study	China	January to September 2020	28 days	239	112	127	Adults ≥ 18 years, first onset, meeting the 2012 international guidelines diagnostic criteria for severe sepsis/septic shock, and no treatment contraindications	ICU stay time, ventilator use time, 28-day mortality, hemodynamic indices, APACHE II and SOFA scores, lactate clearance, complications (ARDS, acute renal failure, MODS), and myocardial injury markers
Jiang et al, 2023 [28]	Retrospective cohort study	China	January 2019 to December 2022	90 days	238	141	97	Age ≥ 18 years, diagnosed with septic shock based on clinical, microbiologic, radiologic, or intraoperative findings, and required IV fluid resuscitation during the acute critical phase	In-hospital mortality
Li et al, 2025 [29]	Retrospective cohort study	China	January 2021 to December 2023	28 dayss	82	41	41	Septic shock per Sepsis-3 SCCM/ESICM definition. Age 18–75 years; normal immune function; APACHE II ≥ 12; crystalloid fluid resuscitation required.	28-day all-cause mortality, fluid infusion volume, and ICU LOS
Rice et al, 2020 [30]	Retrospective cohort study	USA	October 1, 2016, to July 31, 2019	30 days	106	51	55	Adults ≥ 18 years with severe sepsis or septic shock, documented chronic kidney disease (CKD), and ≥ 1 episode of hypotension within 6 h of ED presentation	Total hospital LOS
Zhou et al, 2021 [32]	Retrospective cohort study	China	January 2019 to January 2020	64 days	122	56	66	Clinical diagnosis of sepsis, complete medical records, ethics-approved management, and patient or family consent	Hemodynamic changes (HR, MAP, CVP), cardiac function (CO, SV, LVEF), biomarkers (cTnI, NT-proBNP, CRP), survival rate up to 64 days, and complication incidence
Wang et al, 2021 [31]	Prospective cohort study	China	May 8, 2018, to June 15, 2021	28 days	302	209	93	Age ≥ 18 present with septic shock: infection + SOFA increase ≥ 2, and persistent hypotension needing vasopressors to maintain MAP > 65 mm Hg, and lactate > 2 mmol/L despite fluids	28-day mortality

LOS: length of stay; SIRS: Systemic Inflammatory Response Syndrome; SBP: systolic blood pressure; BP: blood pressure; ED: emergency department; ICU: intensive care unit; IV: intravenous; Sepsis-3: Third International Consensus Definitions for Sepsis and Septic Shock; SCCM: Society of Critical Care Medicine; ESICM: European Society of Intensive Care Medicine; APACHE II: Acute Physiology and Chronic Health Evaluation II; SOFA: Sequential Organ Failure Assessment; ARDS: acute respiratory distress syndrome; MODS: multiple organ dysfunction syndrome; HR: heart rate; MAP: mean arterial pressure; CVP: central venous pressure; CO: cardiac output; SV: stroke volume; LVEF: left ventricular ejection fraction; cTnI: cardiac troponin I; NT-proBNP: N-terminal pro-B-type brain natriuretic peptide; CRP: C-reactive protein.

### Primary outcomes: parallel pairwise meta-analysis

#### Primary effectiveness: all-cause death

**Figure 2:**
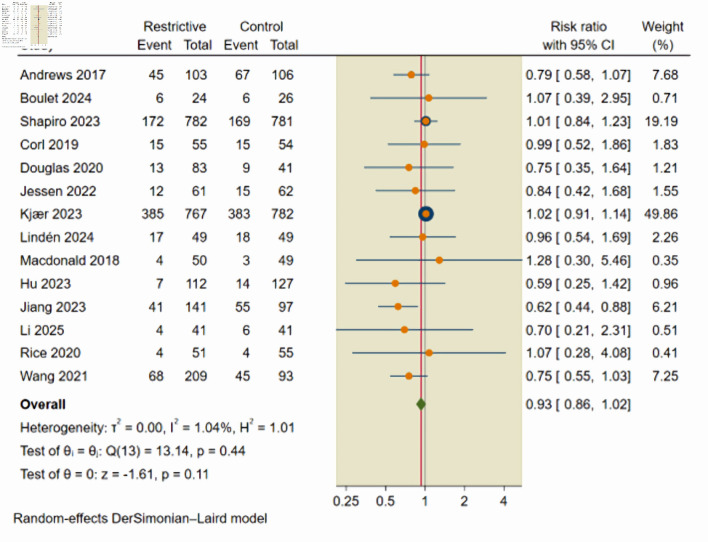
Forest plot of all-cause mortality. CI: confidence interval.

The primary efficacy endpoint was the incidence of all-cause mortality, evaluated across 14 studies that included 2,528 patients who received a restrictive fluid strategy and 2,363 patients who received liberal fluid therapy. The incidence of all-cause mortality was 31.36% in the restrictive fluid group compared with 34.23% in the control groups. The pooled effect estimate using a random-effects model showed no statistically significant difference in all-cause mortality between the two strategies (RR = 0.93; 95% CI, 0.86–1.02; P = 0.11; I^2^ = 1.04%) (Fig. 2).

Given the high risk of confounding in observational data, the primary interpretation of efficacy was based on the analysis of RCTs. In the primary analysis restricted to RCTs (nine studies, n = 3,308), the restrictive fluid strategy resulted in no significant difference in all-cause mortality compared to liberal fluid therapy (RR = 0.99; 95% CI, 0.90–1.08; P = 0.92; I^2^ = 0%), while the analysis of observational studies (five studies, n = 1,583) (Supplementary Material 4, jocmr.elmerjournals.com) demonstrated a significant reduction in mortality associated with restrictive strategies (RR = 0.69; 95% CI, 0.56–0.86; P = 0.001; I^2^ = 0%), which suggests that the apparent mortality benefit seen in observational data reflects confounding by indication, where patients receiving lower fluid volumes were less critically ill at baseline. Consequently, primary conclusion regarding mortality efficacy was based on the RCT subgroup.

Further, L’Abbe plot was used to assess heterogeneity as the plot shows that most studies lie close to the line of no effect, indicating no reduction in mortality risk with restrictive fluid strategies compared with liberal fluid therapy, while larger and more precise studies clustered tightly along this line, reinforcing the overall effect estimate (Supplementary Material 5, jocmr.elmerjournals.com). Additionally, leave-one-out sensitivity analysis (Supplementary Material 6, jocmr.elmerjournals.com) and Galbraith plots (Supplementary Material 7, jocmr.elmerjournals.com) were used to further assess heterogeneity.

**Figure 3:**
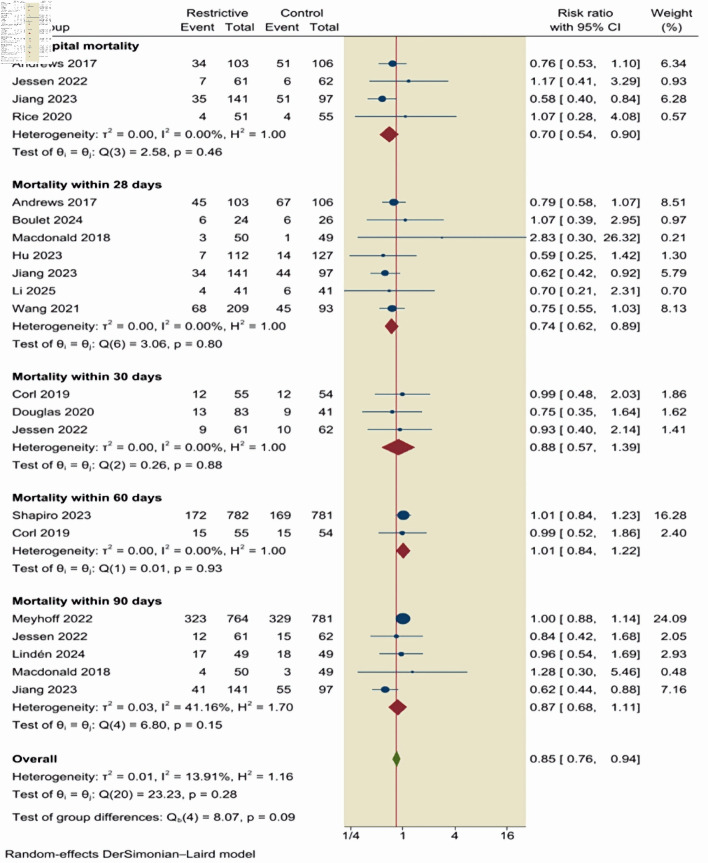
Subgroup analysis of all-cause mortality across multiple follow-up time points. CI: confidence interval.

Subgroup analysis of all-cause mortality across multiple follow-up time points was performed. The pooled estimates demonstrated consistent patterns across categories. However, these findings should be interpreted as exploratory. A restrictive fluid therapy was associated with a statistically significant reduction in in-hospital mortality (RR = 0.70; 95% CI, 0.54–0.90; I^2^ = 0%) and 28-day mortality (RR = 0.74; 95% CI, 0.62–0.89; I^2^ = 0%). No significant differences were observed for 30-day (RR = 0.88; 95% CI, 0.57–1.39; I^2^ = 0%), 60-day (RR = 1.01; 95% CI, 0.84–1.22; I^2^ = 0%), or 90-day mortality (RR = 0.87; 95% CI, 0.68–1.11; I^2^ = 41.16%) (Fig. 3).

**Figure 4:**
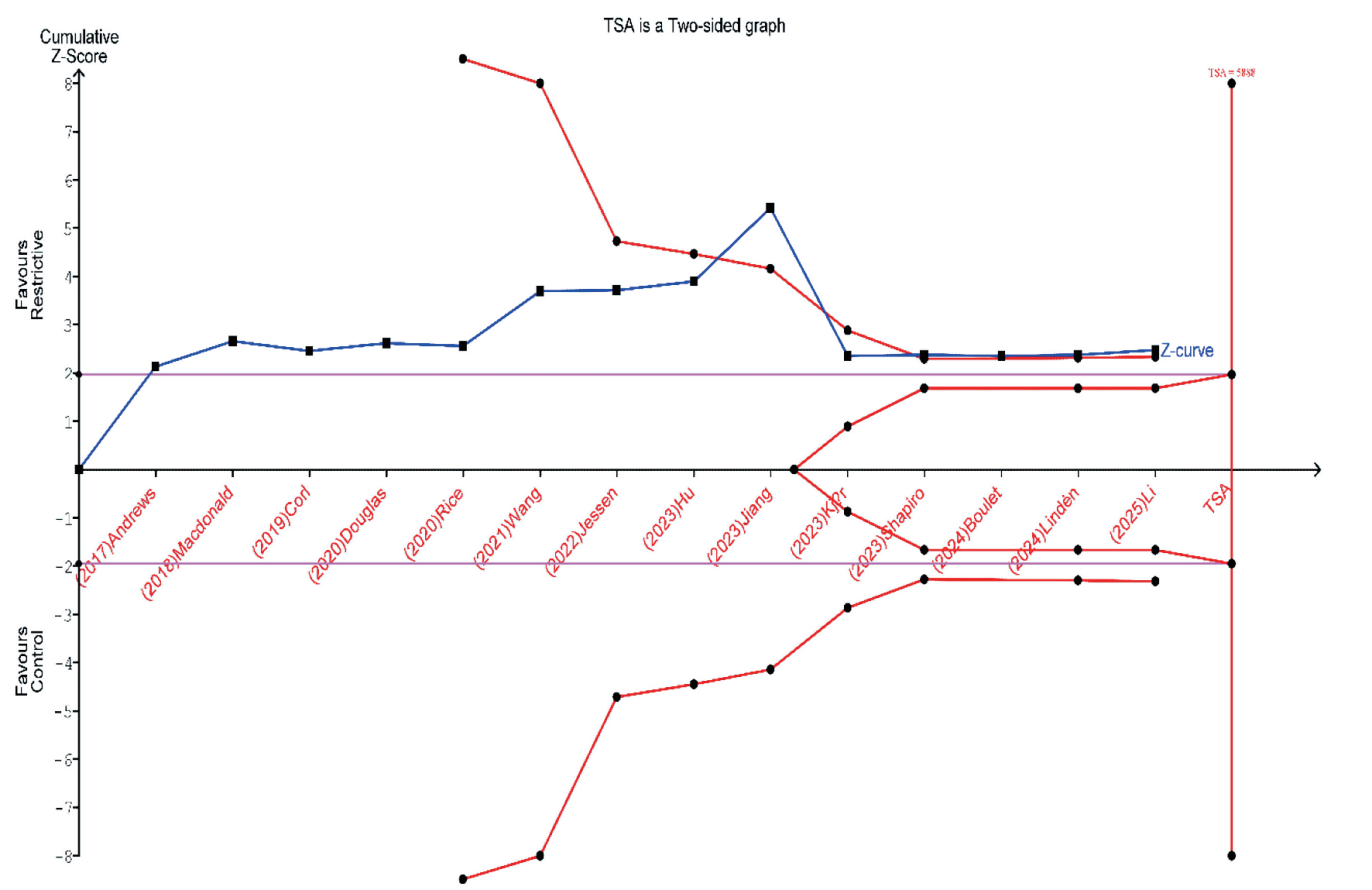
Trial sequential analysis (TSA) for the incidence of all-cause mortality.

TSA for all-cause mortality was conducted using data from 14 studies. The cumulative Z-curve crossed the conventional significance boundary but did not cross the trial sequential monitoring boundary. Although the accrued sample size approached the RIS, the current evidence remains insufficient and inconclusive in determining whether a restrictive fluid strategy reduces all-cause mortality compared to liberal fluid therapy. Accordingly, further large, high-quality, RCTs are needed to establish more definitive conclusions (Fig. 4).

A funnel plot was used to evaluate the potential publication bias. Visual inspection showed that one study lay outside of the estimated effect size (θ_n_), suggesting possible evidence of publication bias (Supplementary Material 8, jocmr.elmerjournals.com).

#### Primary safety outcome: AKI

**Figure 5:**
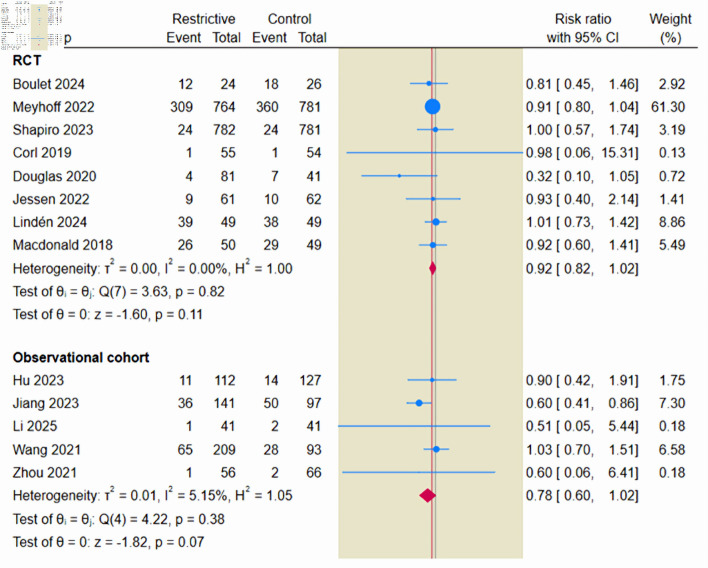
Forest plot of acute kidney injury. RCT: randomized controlled trial; CI: confidence interval.

The primary safety endpoint was the incidence of AKI, based on evidence from 13 studies. The incidence of AKI was 22.1% in the restrictive fluid group (538/2,425) and 25.7% in the control group (583/2,267). The pooled estimate demonstrated a reduction in the incidence of AKI with the restrictive fluid strategy compared to the control group (RR = 0.89; 95% CI, 0.81–0.99; P = 0.02; I^2^ = 0%) (Fig. 5).

When restricting the analysis to RCTs only, the signal for renal safety remained consistent; the restrictive strategy was associated with a reduced risk of AKI (RR = 0.92; 95% CI, 0.85–0.99), driven by high-weight trials such as CLASSIC and CLOVERS.

**Figure 6:**
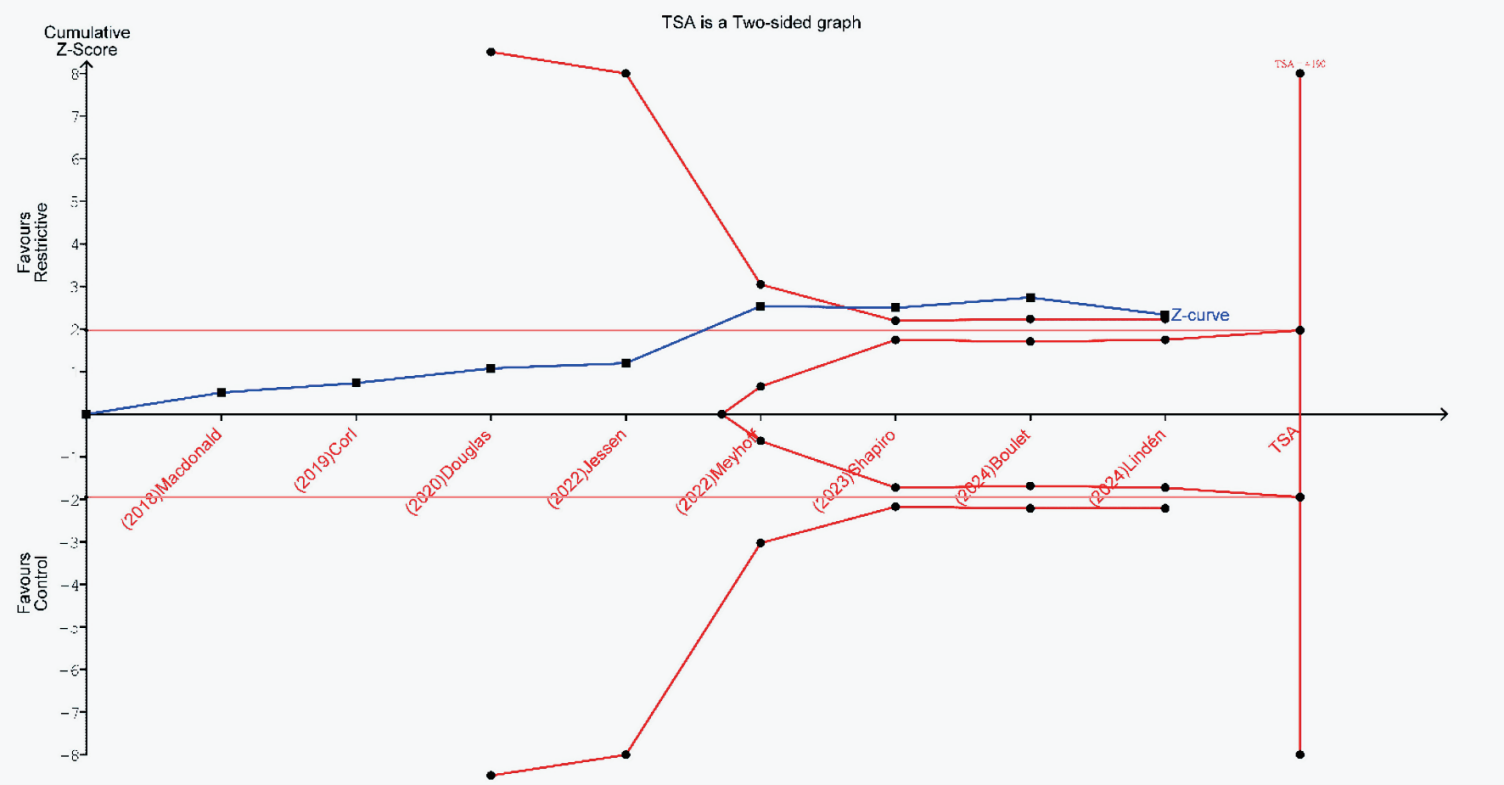
Trial sequential analysis (TSA) for the incidence of acute kidney injury.

Leave-one-out sensitivity analysis demonstrated that the overall pooled effect for AKI remained stable and statistically significant after the sequential removal of each study. However, after the exclusion of the study by Meyhoff et al (2022) [13] or Jiang et al (2023) [28], the pooled estimate was not significant (Supplementary Material 9, jocmr.elmerjournals.com). Additionally, a subgroup analysis stratified by AKI stage showed no significant differences between the restrictive and control fluid strategies for either AKI stage 2 (RR = 0.97; 95% CI, 0.62–1.51; P = 0.67; I^2^ = 0%) or AKI stage 3 (RR = 0.84; 95% CI, 0.48–1.47; P = 0.41; I^2^ = 0%) (Supplementary Material 10, jocmr.elmerjournals.com). TSA was performed to assess the effect of restrictive fluid therapy on the risk of AKI based on evidence from 13 studies. Although the cumulative Z-curve crossed the conventional monitoring boundary, it did not cross the sequential trial monitoring boundary, which indicates that the current evidence is insufficient and inconclusive, underscoring the need for large-scale randomized trials to validate this finding (Fig. 6).

### Secondary outcomes: parallel pairwise meta-analysis

#### Clinical and safety outcomes

There was no significant difference between the restrictive fluid group and the control group regarding ischemic events, including cerebral ischemia (RR = 1.01; 95% CI, 0.55–1.88; P = 0.98; I^2^ = 0%), intestinal ischemia (RR = 0.94; 95% CI, 0.62–1.41; P = 0.82; I^2^ = 0%), limb ischemia (RR = 1.04; 95% CI, 0.57–1.90; P = 0.91; I^2^ = 0%), or myocardial ischemia (RR = 2.02; 95% CI, 0.95–4.27; P = 0.61; I^2^ = 0%) (Supplementary Material 11, jocmr.elmerjournals.com). Moreover, there was no notable variation observed between the two fluid strategies regarding DIC (RR = 0.44; 95% CI, 0.08–2.27; P = 0.32; I^2^ = 0%), MODS (RR = 0.78; 95% CI, 0.44–1.39; P = 0.41; I^2^ = 0%), ICU admission rate (RR = 1.07; 95% CI, 0.98–1.17; P = 0.15; I^2^ = 0%), pulmonary edema (RR = 0.49; 95% CI, 0.08–2.94; P = 0.44; I^2^= 0%), or total serious adverse events (RR = 0.94; 95% CI, 0.82–1.09; P = 0.45; I^2^ = 0%) (Supplementary Materials 12–16, jocmr.elmerjournals.com). In contrast, the incidence of ARDS was reported in five studies, including 1,200 patients in the restrictive fluid group and 1,108 in the control group. The pooled analysis demonstrated that patients receiving a restrictive fluid strategy had a significantly lower incidence of ARDS compared with those receiving liberal fluid (RR = 0.69; 95% CI, 0.56–0.85; P < 0.001; I^2^ = 0%) (Supplementary Material 17, jocmr.elmerjournals.com).

#### Electrolyte and metabolic outcomes

No significant differences were observed between the restrictive and liberal fluid groups in terms of metabolic or electrolyte disturbances, including hyperchloremia (RR = 0.81; 95% CI, 0.38–1.75; P = 0.98; I^2^ = 0%), hypernatremia (RR = 0.89; 95% CI, 0.51–1.54; P = 0.72; I^2^ = 0%), hyponatremia (RR = 1.06; 95% CI, 0.11–9.85; P = 0.98; I^2^ = 0%), or hypoglycemia (RR = 2.47; 95% CI, 0.88–6.93; P = 0.62; I^2^ = 0%) (Supplementary Material 18, jocmr.elmerjournals.com).

#### Secondary efficacy outcomes

Across additional secondary outcomes, restrictive fluid management showed no significant difference regarding hospital length of stay (MD = 0.02; 95% CI, -1.33 to 1.37; P = 0.97; I^2^ = 68.16), ICU length of stay (MD = -0.31; 95% CI, -0.97 to 0.36; P = 0.37; I^2^ = 72.43), organ support-free days (MD = 0.11; 95% CI, -0.46 to 0.69; P = 0.70; I^2^ = 0) or vasopressor-free days (MD = 1.42; 95% CI, -0.33 to 3.16; P = 0.11; I^2^ = 73.56) (Supplementary Material 19–22, jocmr.elmerjournals.com). Similarly, no significant differences were observed in vasopressin use (RR = 0.65; 95% CI, 0.30–1.44; P = 0.29; I^2^ = 65.87%), initiation of RRT (RR = 0.85; 95% CI, 0.65–1.10; P = 0.21; I^2^ = 28.94%) compared with liberal fluid therapy (Supplementary Materials 23, 24, jocmr.elmerjournals.com). In contrast, the restrictive fluid strategy was associated with a significant reduction in the need for mechanical ventilation (Supplementary Material 25, jocmr.elmerjournals.com) (RR = 0.82; 95% CI, 0.71–0.94; P < 0.001; I^2^ = 0%), duration of mechanical ventilation (MD = -1.38; 95% CI, -2.36 to -0.41; P = 0.01; I^2^ = 56.55), and duration of vasopressin use (MD = -8.90; 95% CI: -15.80 to -2; P = 0.01; I^2^ = 0) (Supplementary Material 26, 27, jocmr.elmerjournals.com). Additionally, restrictive fluid management significantly increased ventilation-free days (MD = 2.12; 95% CI, 0.35–3.89; P = 0.02; I^2^ = 80.43) (Supplementary Material 28, jocmr.elmerjournals.com).

## Discussion

### Summary of key findings

This systematic review and meta-analysis synthesize evidence comparing restrictive and liberal fluid strategies in adults with sepsis and septic shock. The analysis indicates that a restrictive fluid strategy is associated with clinical benefits, specifically a reduced risk of AKI and a lower incidence of ARDS. Also, patients managed with a restrictive approach exhibited a decreased need for mechanical ventilation, shorter durations of mechanical ventilation and vasopressor support, and an increase in ventilator-free days compared with those receiving liberal fluid therapy. Despite these benefits, the restrictive strategy did not reduce the primary outcome of overall all-cause mortality in the primary RCT analysis. While subgroup analyses revealed a significant difference in in-hospital and 28-day mortality. These early time-point findings must be viewed with caution due to the lack of signal in the primary long-term analysis.

Additional subgroup analyses indicated that observational studies showed a greater mortality benefit than RCTs. TSA suggests that the current evidence remains insufficient to draw definitive conclusions regarding mortality and AKI reduction. No significant differences were observed between the two strategies regarding ischemic events (cerebral, intestinal, limb, or myocardial), metabolic or electrolyte disturbances (hyperchloremia, hypernatremia, hyponatremia, and hypoglycemia), initiation of RRT, ICU or hospital length of stay, organ support-free days, vasopressor-free days, or other serious adverse events.

### Interpretations of main findings

The primary finding of our meta-analysis is that a restrictive fluid strategy does not significantly reduce long-term all-cause mortality compared to liberal standard care, which aligns with the results of several recent large-scale RCTs and prior meta-analyses [13, 14, 35, 36]. This comparable mortality outcome may be attributed to the evolving standard of care, wherein clinicians in the liberal/control groups of modern trials have increasingly adopted more conservative fluid administration practices compared with historical cohorts (the Hawthorne effect), thereby narrowing the volume separation between study groups [22, 37]. Furthermore, sepsis is a heterogeneous syndrome with varying phenotypes; a one-size-fits-all restrictive approach may benefit patients with a congestive phenotype while potentially harming those with true hypovolemia, diluting the overall mortality signal [38, 39].

However, subgroup analysis identified a significant reduction in in-hospital and 28-day mortality, which contrasts with the findings of Reynolds et al [40], who reported no significant mortality benefit at any time point. This early mortality benefit in the current study stems from the mitigation of early iatrogenic fluid overload, which exacerbates tissue edema and organ dysfunction during the critical inflammatory phase of sepsis [41, 42].

The discrepancy between early survival benefits and neutral long-term (90-day) outcomes suggests that while fluid restriction may prevent early death from refractory shock or respiratory failure, long-term survival is driven by non-hemodynamic factors such as immunosuppression, post-intensive care syndrome, and underlying comorbidities [13]. Also, the analysis indicates that observational studies showed a greater mortality benefit than RCTs, suggesting confounding by indication in the non-randomized cohorts. In real-world practice, patients receiving liberal fluids are often those presenting with more severe shock or hypovolemia, thereby inflating the mortality rate of the control group artificially [13].

Regarding renal outcomes, the analysis demonstrated a significant reduction in the risk of AKI with the restrictive strategy, which corroborates the recent work by Cai et al, who concluded that fluid restriction is superior in preventing severe AKI [43]. This finding is robust when considering the potential for dilutional bias in fluid trials, as liberal fluid administration leads to hemodilution, which artificially lowers serum creatinine concentrations, masking the diagnosis of AKI. Conversely, restrictive strategies may lead to hemoconcentration, increasing the AKI diagnosis based on creatinine criteria. The restrictive group demonstrated a lower incidence of AKI despite a detection bias that favors the liberal group; this supports the physiological argument that fluid restriction prevents renal venous congestion and kidney injury [44–47]. This finding diverges from those of Reynolds et al [40] and Duan et al [48], who found no significant difference in AKI or RRT rates. This variance may be attributed to the inclusion of newer trials that implemented stricter fluid limitation protocols and responsiveness testing, thereby preventing the congestive kidney failure often seen in liberal groups [25, 34].

In terms of respiratory outcomes, the restrictive strategy was associated with a lower incidence of ARDS, a reduced need for mechanical ventilation, and an increase in ventilator-free days, which algin with the findings of Shahnoor et al [49], who reported shorter durations of mechanical ventilation in restrictive ventilation groups. The physiological mechanism is attributed to the preservation of the endothelial glycocalyx and the prevention of pulmonary hydrostatic edema [50, 51]. In sepsis, increased capillary permeability makes the lungs susceptible to fluid extravasation, impairing gas exchange and lung compliance [52, 53]. Iatrogenic pulmonary edema is minimized by limiting hydrostatic pressure through fluid restriction, facilitating earlier liberation from mechanical ventilation [44, 54].

Also, a significant reduction in vasopressor support duration in the restrictive group was observed. This contrasts with the theoretical concern that fluid restriction might require prolonged vasopressor use to maintain mean arterial pressure in the absence of volume loading [33, 34]. The reduction in vasopressor duration may be explained by the minimization of tissue edema, which can impair microcirculatory perfusion and oxygen extraction, perpetuating cellular shock and the need for hemodynamic support [55]. Additionally, restrictive protocols often mandate the earlier initiation of vasopressors (e.g., norepinephrine), which may restore vascular tone and recruit unstressed blood volume more than crystalloids alone, leading to faster shock resolution [26, 56–58]. The lack of significant differences in ischemic events (cerebral, myocardial, or intestinal) or metabolic disturbances in the analysis suggests that restrictive strategies, when guided by safety limits, do not compromise end-organ perfusion, supporting the safety profile reported in the CLASSIC and CLOVERS trials [13, 14].

### Strengths and limitations

This systematic review and meta-analysis had several methodological strengths that enhanced the validity of the findings. The inclusion of a large sample size comprising 5,013 patients from 15 unique studies increases the statistical power to detect significant differences in secondary outcomes such as AKI and mechanical ventilation, addressing the sample size limitations in previous individual trials. Also, TSA provides a statistical adjustment for repeated significance testing and evaluates the conclusiveness of the evidence, preventing premature conclusions regarding mortality benefits, a methodological rigor absent in many previous meta-analyses.

Extensive subgroup analyses were incorporated, including stratification by AKI stage and follow-up duration to clarify the discrepancy between short-term physiological benefits and long-term survival outcomes. In addition, the search strategy encompassing RCTs and observational studies allowed for a broader evaluation of effectiveness versus efficacy in controlled settings, highlighting the divergence in results between study designs.

However, several limitations should be acknowledged such as the significant clinical and methodological heterogeneity in the definition of restrictive and liberal strategies. Protocols varied significantly, ranging from absolute volume caps (e.g., ≤ 60 mL/kg, the FEAST-style fluid sparing) to dynamic responsiveness-guided restrictions (e.g., passive leg raise), complicating the isolation of the specific element of care responsible for improved outcomes. Despite this clinical heterogeneity, the statistical heterogeneity (I^2^) for outcomes such as AKI and ARDS remained low (0%), as the direction of the effect (favoring restriction for safety outcomes) was consistent across trials, even if the magnitude varied. The meta-analysis of mortality was dominated by a single large trial (Kjaer/CLASSIC), which contributed half of the statistical weight in the RCT subgroup. While this increases the precision of the estimate, it also means the result is reflective of this specific protocol, which compared a restrictive strategy against a standard care group that had already adopted relatively conservative fluid practices compared to historical controls. Furthermore, in major RCTs, such as CLOVERS and CLASSIC, patients in the restrictive group received substantial volumes of fluid prior to randomization (pre-enrolment fluids). However, we did not exclude these trials via sensitivity analysis because they represent the highest-quality and most pragmatic evidence currently available, reflecting the real-world standard where safety boluses precede protocolized restriction. While this may have diluted the separation between groups, excluding them would compromise the external validity and statistical power of the meta-analysis. Additionally, the inclusion of observational studies introduces the potential for selection bias and confounding by indication, as sicker patients are more likely to receive liberal fluid resuscitation, skewing mortality results in favor of restrictive strategies in non-randomized cohorts.

### Clinical implications and advances in knowledge

The findings of this meta-analysis have substantial implications for the clinical management of sepsis and septic shock. The reduction in AKI and ARDS with restrictive fluid strategies challenges the traditional paradigm that aggressive volume loading is necessary to preserve organ perfusion. Instead, the data support a shift toward fluid stewardship, suggesting that preventing iatrogenic salt and water overload is as critical as initial resuscitation in preventing congestive kidney failure and pulmonary edema. Clinicians should adopt a conservative approach, particularly after the initial resuscitation phase, prioritizing vasopressors and dynamic assessment over empiric fluid boluses to minimize the duration of mechanical ventilation and ICU stay. Furthermore, the safety profile of ischemic events suggests that restrictive strategies do not compromise macrovascular perfusion to critical organs, alleviating concerns about inducing end-organ ischemia.

Future research should move to precision medicine as there is a critical need to identify specific sepsis phenotypes (e.g., those with high vascular permeability vs. hypovolemia) that may benefit differentially from specific fluid strategies. Additionally, future trials should focus on the pre-hospital or immediate emergency department phase to minimize pre-randomization fluid administration, thereby testing the true efficacy of early restrictions. Finally, future studies should include long-term patient-centered outcomes such as cognitive function and health-related quality of life, rather than focusing on short-term mortality because survival is often influenced by post-acute factors.

### Conclusions

Restrictive fluid resuscitation yields comparable long-term all-cause mortality to that of liberal standard care in adults with sepsis, yet it demonstrates superior clinical efficacy by reducing the incidence of AKI and ARDS, as well as shortening the duration of mechanical ventilation and vasopressor support. Restrictive fluid stewardship represents a preferable standard of care for mitigating iatrogenic organ dysfunction without compromising tissue perfusion because of its favorable safety profile with no increase in ischemic events. Clinicians should prioritize this approach to avoid fluid overload, and future research should focus on identifying specific sepsis phenotypes that benefit from precision resuscitation strategies.

## Supplementary Material

Suppl 1 Search strategy for each database.

Suppl 2 Risk of bias assessment for RCTs by ROB-2.

Suppl 3 Risk of bias assessment for observational studies by the Newcastle–Ottawa Scale.

Suppl 4 Subgroup analysis of all-cause death stratified by study design.

Suppl 5 L’Abbe plot of all-cause death.

Suppl 6 Leave-one-out sensitivity analysis of all-cause death.

Suppl 7 Galbraith plot of all-cause death.

Suppl 8 Funnel plot of all-cause death.

Suppl 9 Leave-one-out sensitivity analysis of acute kidney injury.

Suppl 10 Subgroup analysis of acute kidney injury stratified by AKI stage.

Suppl 11 Forest plot of ischemic events, including cerebral ischemia, intestinal ischemia, limb ischemia, and myocardial ischemia.

Suppl 12 Forest plot of disseminated intravascular coagulopathy (DIC).

Suppl 13 Forest plot of multiple organ dysfunction syndrome (MODS).

Suppl 14 Forest plot of intensive care unit (ICU) admission rate.

Suppl 15 Forest plot of pulmonary edema.

Suppl 16 Forest plot of total serious adverse events

Suppl 17 Forest plot of incidence of acute respiratory distress syndrome.

Suppl 18 Forest plot of electrolyte and metabolic outcomes, including hyperchloremia, hypernatremia, hyponatremia, and hypoglycemia.

Suppl 19 Forest plot of hospital length of stay (days).

Suppl 20 Forest plot of ICU length of stay (days).

Suppl 21 Forest plot of organ support-free days.

Suppl 22 Forest plot of vasopressor-free days.

Suppl 23 Forest plot of vasopressin use.

Suppl 24 Forest plot of initiation of renal replacement therapy.

Suppl 25 Forest plot of the need for mechanical ventilation.

Suppl 26 Forest plot of duration of mechanical ventilation (days).

Suppl 27 Forest plot of duration of vasopressin support (hours).

Suppl 28 Forest plot of ventilation-free days.

## Data Availability

All data generated or analyzed during this study are included in this published article and its Supplementary information files. The search strategies and data extraction forms are available from the corresponding author upon reasonable request.

## Abbreviations

AKI: acute kidney injury
ARDS: acute respiratory distress syndrome
RR: risk ratio
MD: mean difference
TSA: trial sequential analysis
RCTs: randomized controlled trials
MOOSE: Meta-Analysis of Observational Studies in Epidemiology
ROB-2: Risk Of Bias-2
NOS: Newcastle–Ottawa Scale
SSC: Surviving Sepsis Campaign
RRR: renal replacement therapy
DIC: disseminated intravascular coagulation
MODS: multiple organ dysfunction syndrome
ICU: intensive care unit
