# Supplementary material for: Restrictive Versus Liberal Fluid Strategy for Initial Resuscitation in Sepsis and Septic Shock: A Systematic Review and Meta Analysis
Source: J Clin Med Res. 2026 Mar 26;18(3):177–95. doi: 10.14740/jocmr6464 (PMC13053473; doi:10.14740/jocmr6464)
Supplement: Suppl 1 — Search strategy for each database. [file jocmr-18-03-177-s001.docx]

**Suppl 1.** Search strategy for each database

| Database | Search term | Filter | Results |
| --- | --- | --- | --- |
| PubMed | (("sepsis" OR "septic shock") AND (“restrictive fluid" OR “fluid sparing" OR "liberal" OR "usual care" OR "standard care")) | All Filed | 863 |
| Scopus | (("sepsis" OR "septic shock") AND (“restrictive fluid" OR “fluid sparing" OR "liberal" OR "usual care" OR "standard care")) | Article title, abstract, keywords | 1133 |
| Web of Science | (("sepsis" OR "septic shock") AND (“restrictive fluid" OR “fluid sparing" OR "liberal" OR "usual care" OR "standard care")) | All Filed | 920 |
| Cochrane library | (("sepsis" OR "septic shock") AND (“restrictive fluid" OR “fluid sparing" OR "liberal" OR "usual care" OR "standard care")) | All Filed | 725 |
