# Supplementary figures and images for: Restrictive Versus Liberal Fluid Strategy for Initial Resuscitation in Sepsis and Septic Shock: A Systematic Review and Meta Analysis
Source: J Clin Med Res. 2026 Mar 26;18(3):177–95. doi: 10.14740/jocmr6464 (PMC13053473; doi:10.14740/jocmr6464)

**Suppl 2.** Risk of bias assessment for RCTs by ROB-2.

**
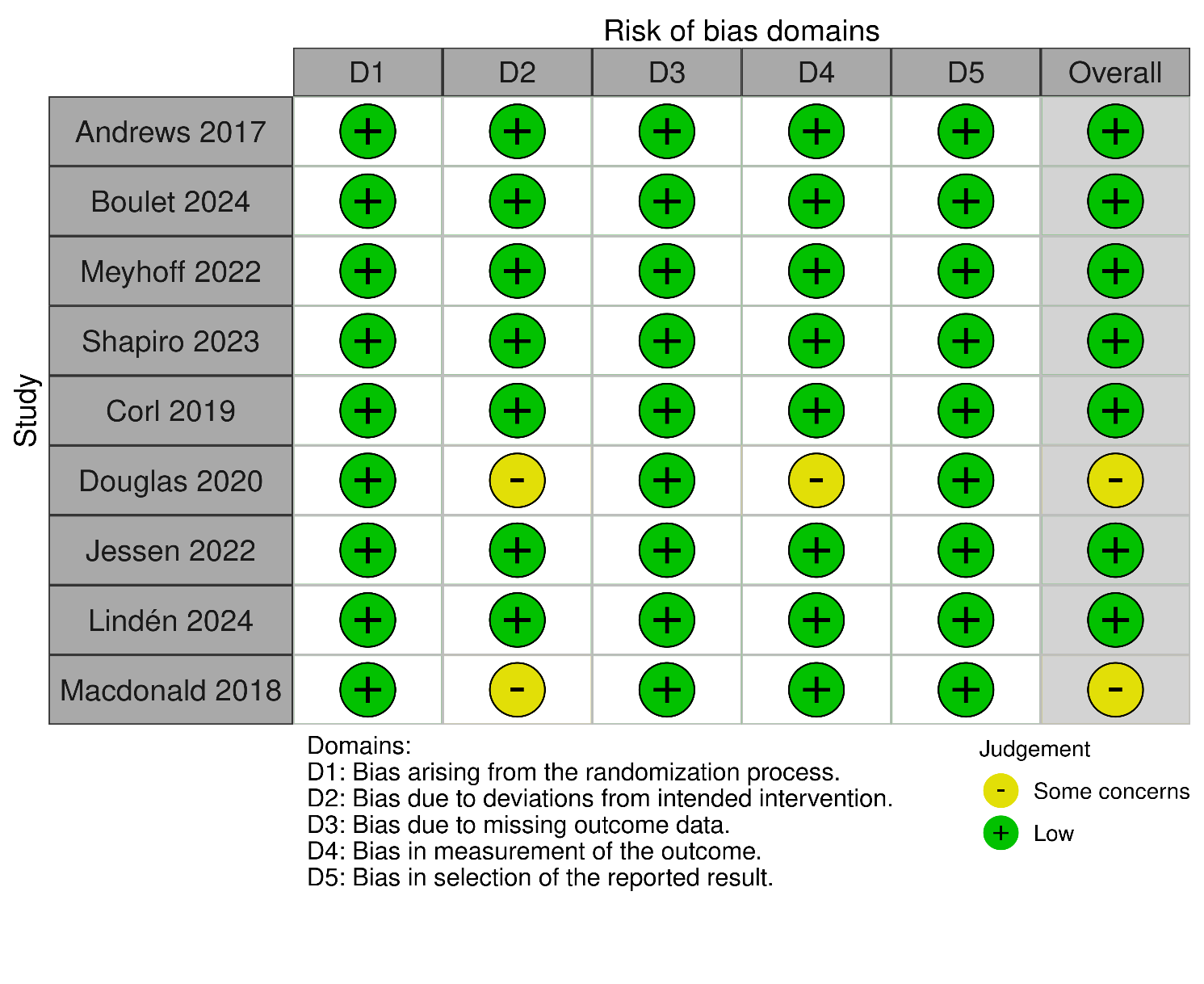
**

Supplement: Suppl 2 — Risk of bias assessment for RCTs by ROB-2. [file jocmr-18-03-177-s002.docx]

**Suppl 3.** Risk of bias assessment for observational studies by the Newcastle–Ottawa Scale.
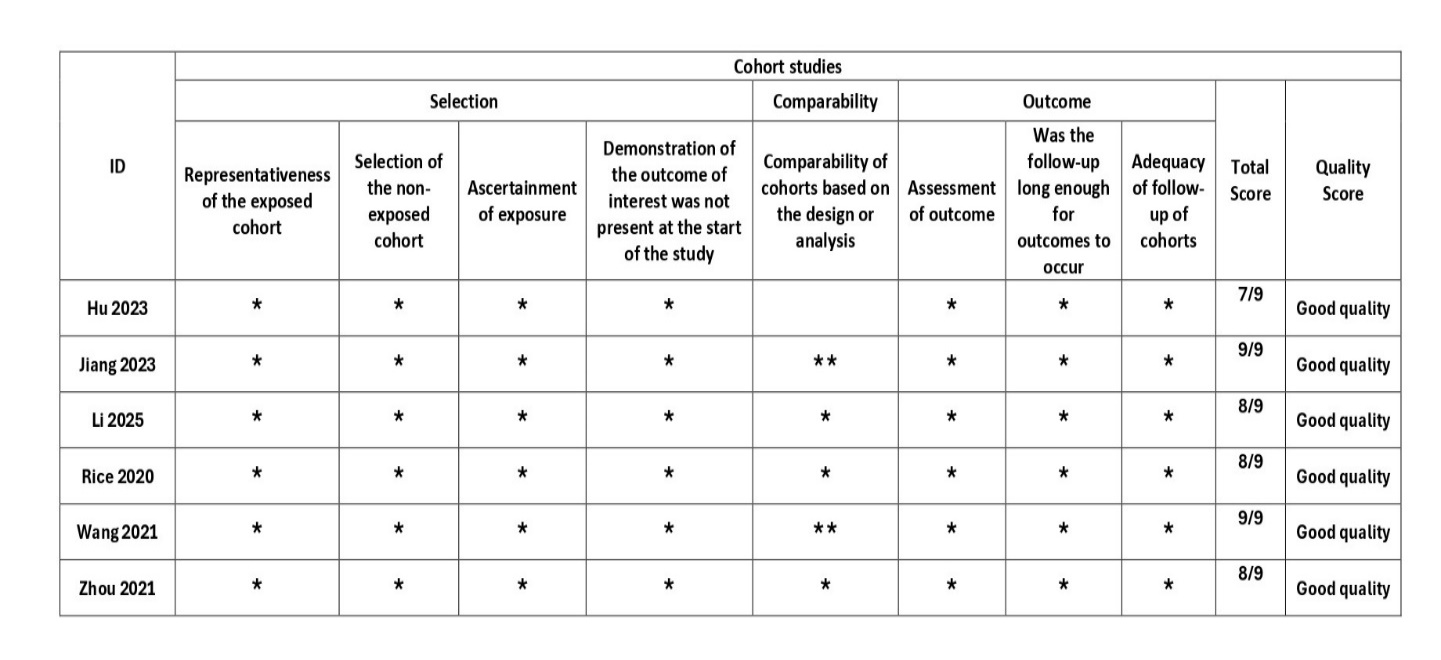

Supplement: Suppl 3 — Risk of bias assessment for observational studies by the Newcastle–Ottawa Scale. [file jocmr-18-03-177-s003.docx]

**Suppl 4.** Subgroup analysis of all-cause death stratified by study design.

**
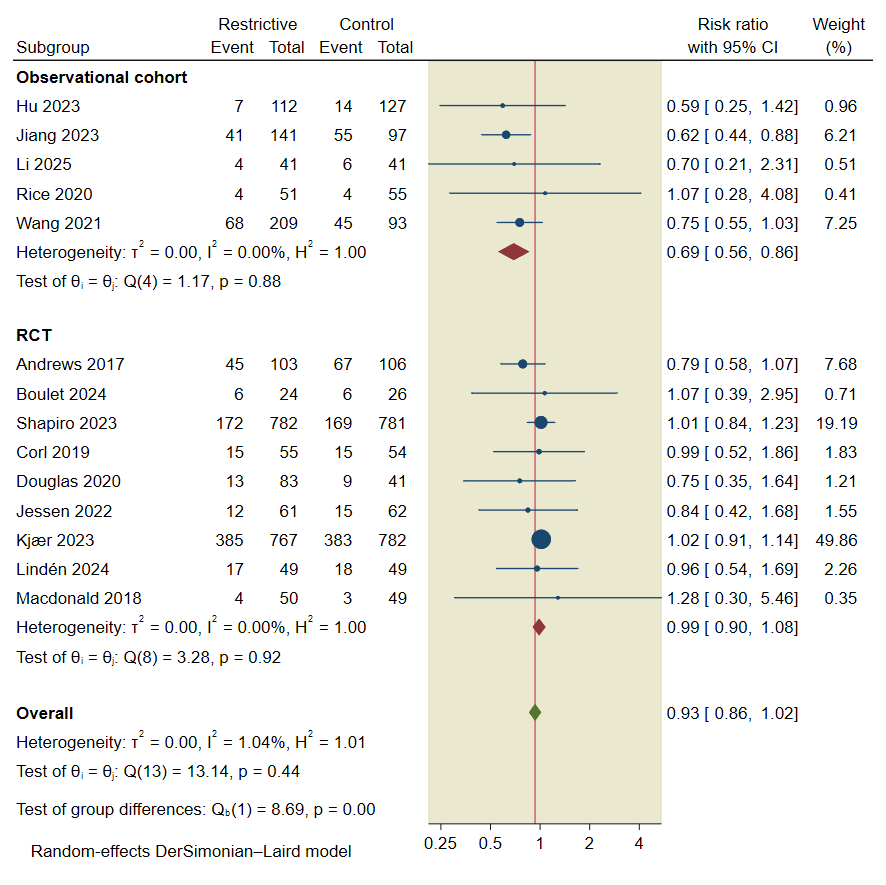
**

Supplement: Suppl 4 — Subgroup analysis of all-cause death stratified by study design. [file jocmr-18-03-177-s004.docx]

**Suppl 5.** L’Abbé plot of all-cause death

**
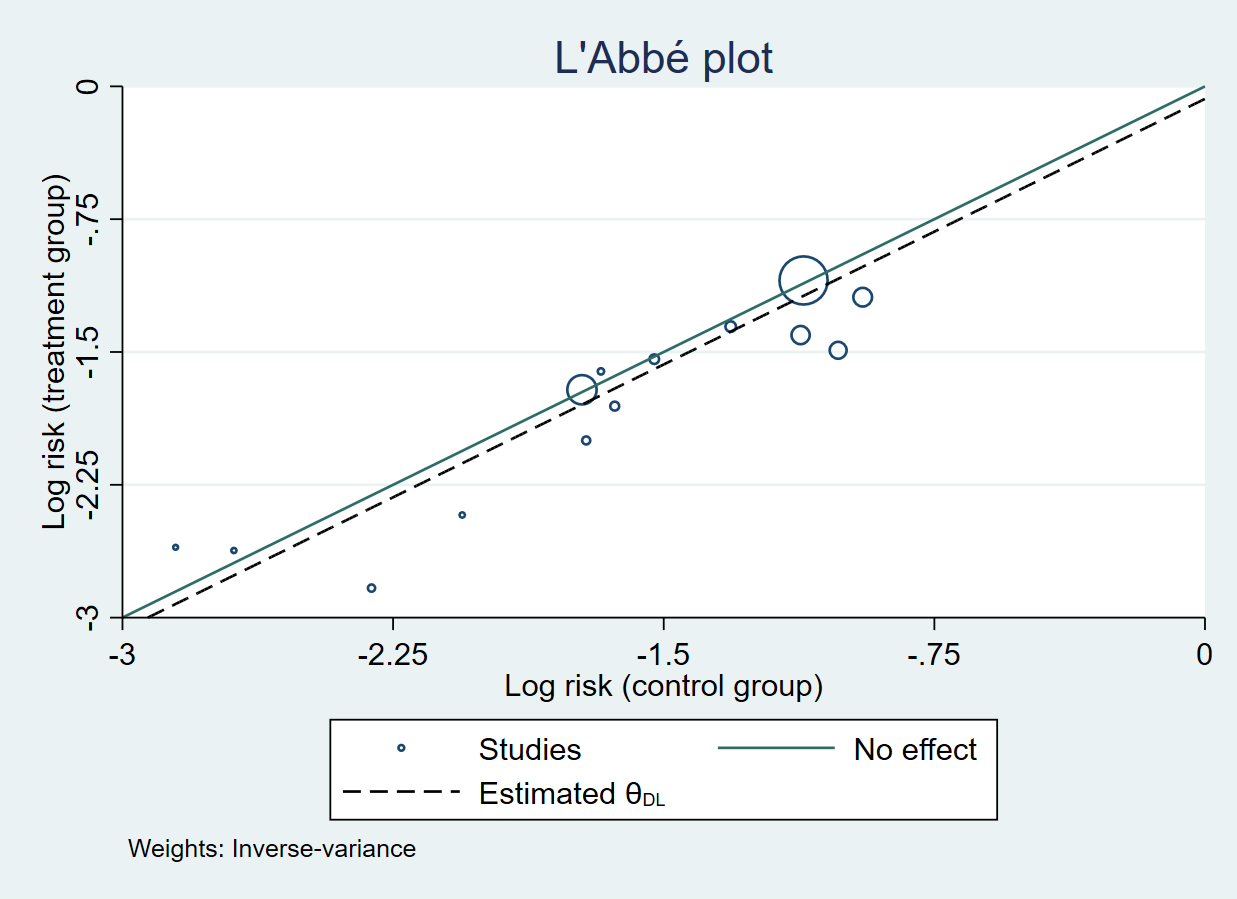
**

Supplement: Suppl 5 — L’Abbe plot of all-cause death. [file jocmr-18-03-177-s005.docx]

**Suppl 6.** Leave-one-out sensitivity analysis of all-cause death

**
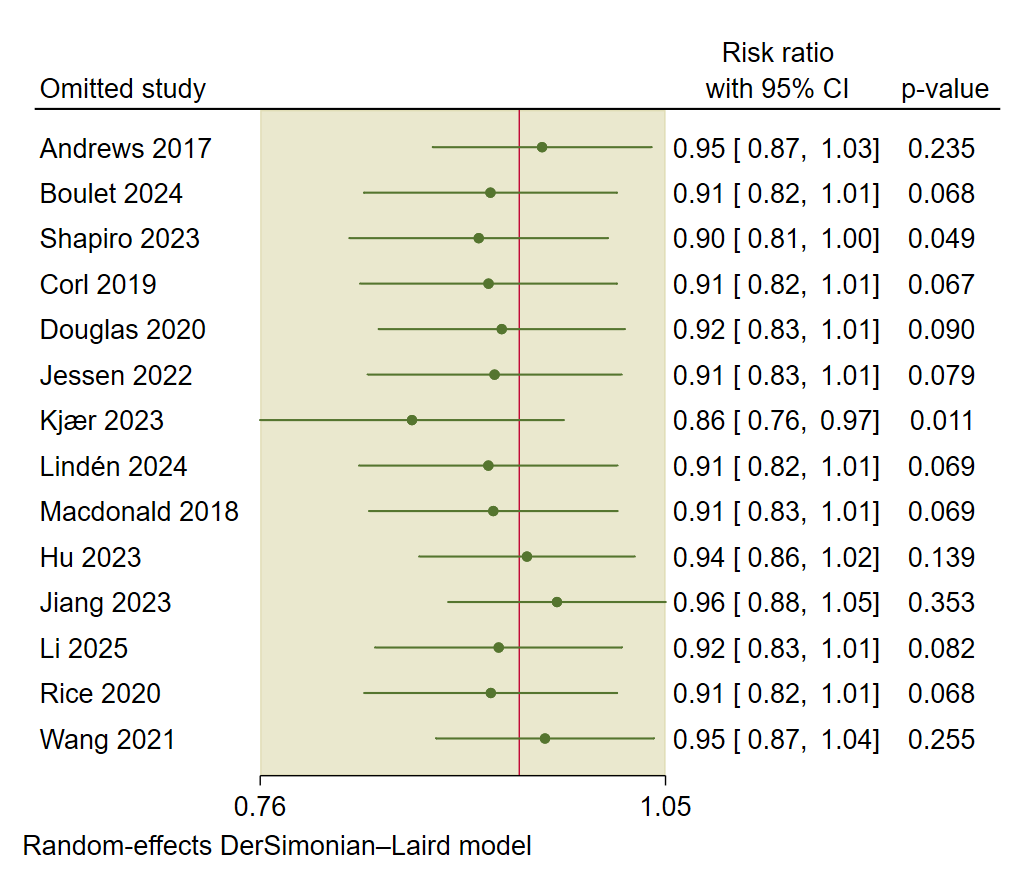
**

Supplement: Suppl 6 — Leave-one-out sensitivity analysis of all-cause death. [file jocmr-18-03-177-s006.docx]

**Suppl 7.** Galbraith plot of all-cause death

**
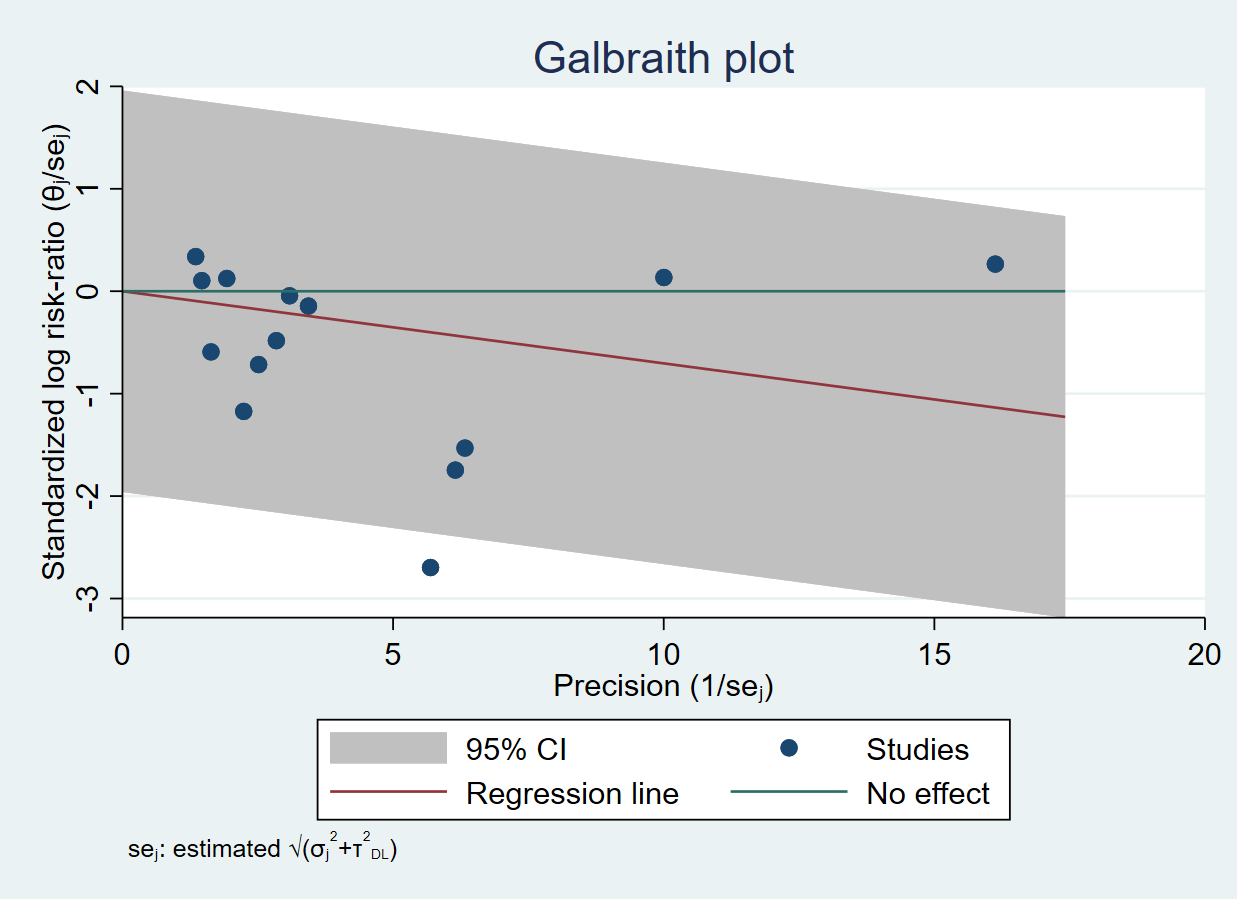
**

Supplement: Suppl 7 — Galbraith plot of all-cause death. [file jocmr-18-03-177-s007.docx]

**Suppl 8.** Funnel plot of all-cause death

**
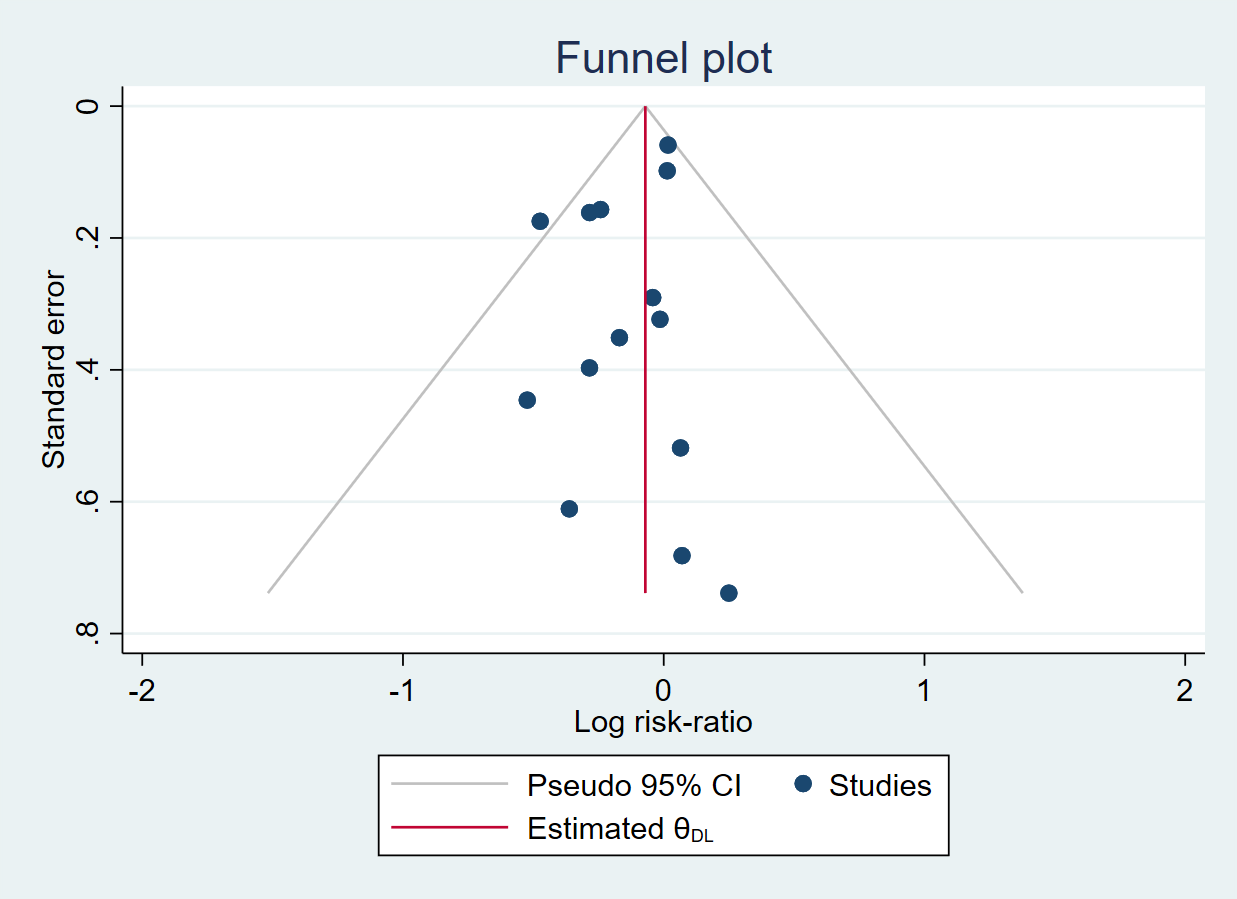
**

Supplement: Suppl 8 — Funnel plot of all-cause death. [file jocmr-18-03-177-s008.docx]

**Suppl 9.** Leave-one-out sensitivity analysis of acute kidney injury


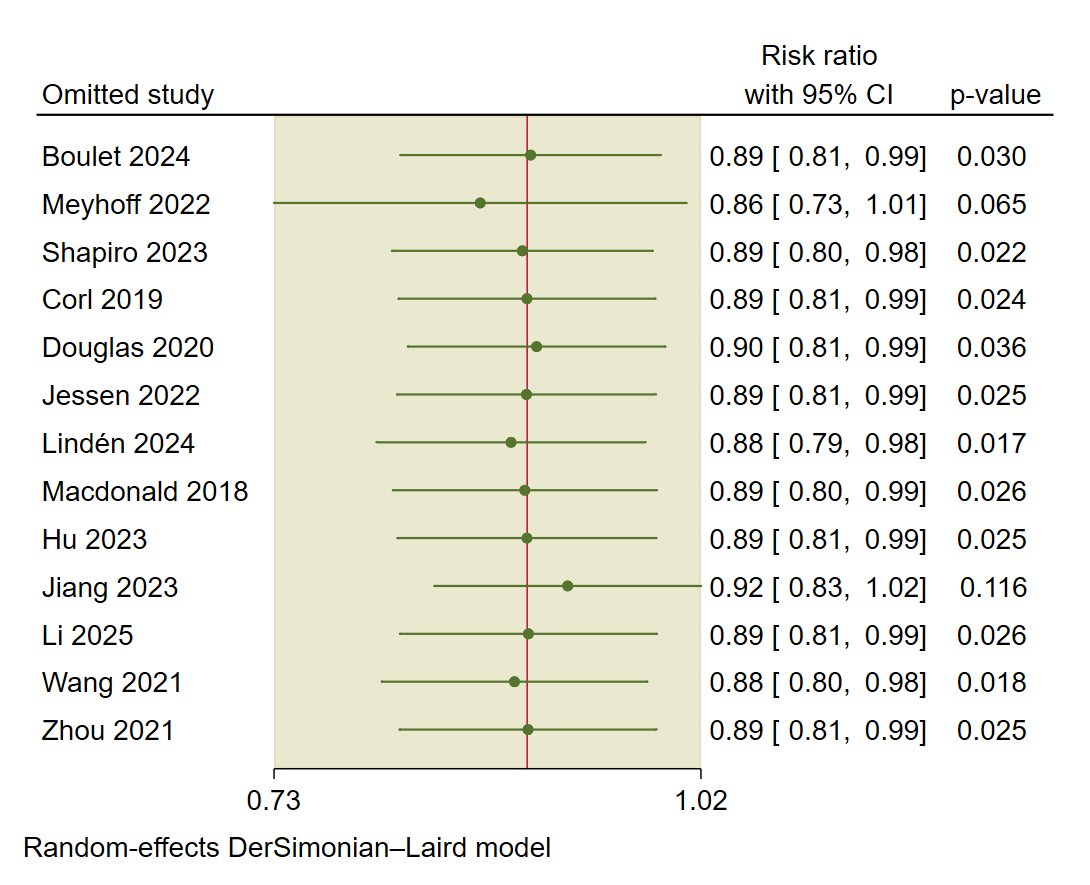

Supplement: Suppl 9 — Leave-one-out sensitivity analysis of acute kidney injury. [file jocmr-18-03-177-s009.docx]

**Suppl 10.** Subgroup analysis of acute kidney injury stratified by AKI stage


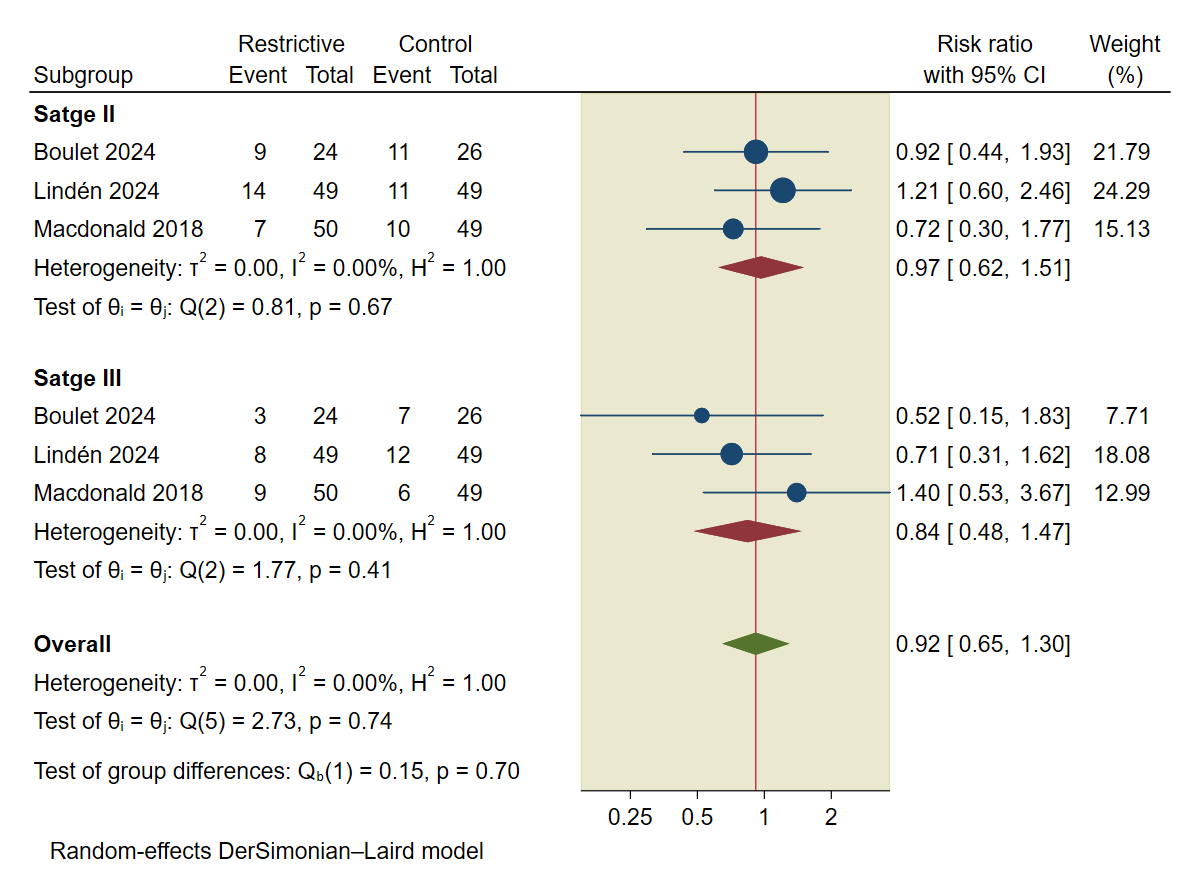

Supplement: Suppl 10 — Subgroup analysis of acute kidney injury stratified by AKI stage. [file jocmr-18-03-177-s010.docx]

**Suppl 12.** Forest plot of disseminated intravascular coagulopathy (DIC)


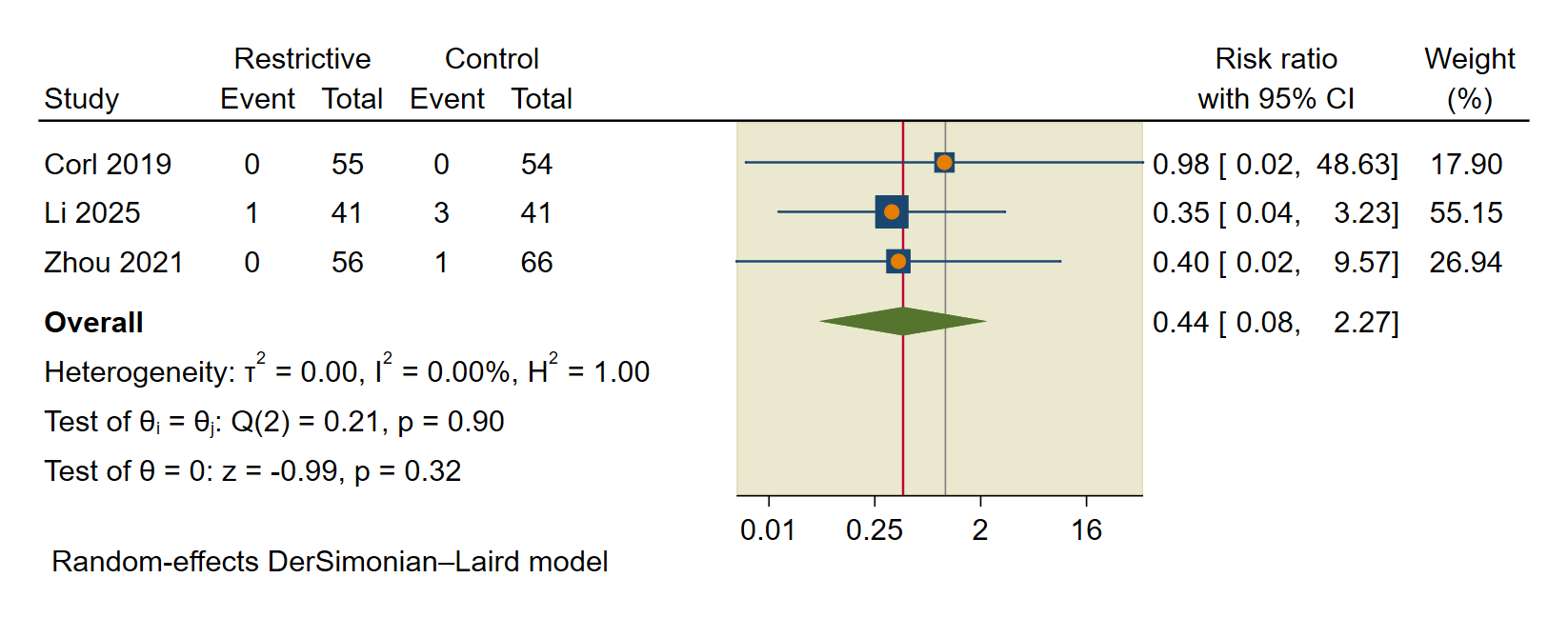

Supplement: Suppl 12 — Forest plot of disseminated intravascular coagulopathy (DIC). [file jocmr-18-03-177-s012.docx]

**Suppl 13.** Forest plot of multiple organ dysfunction syndrome (MODS)


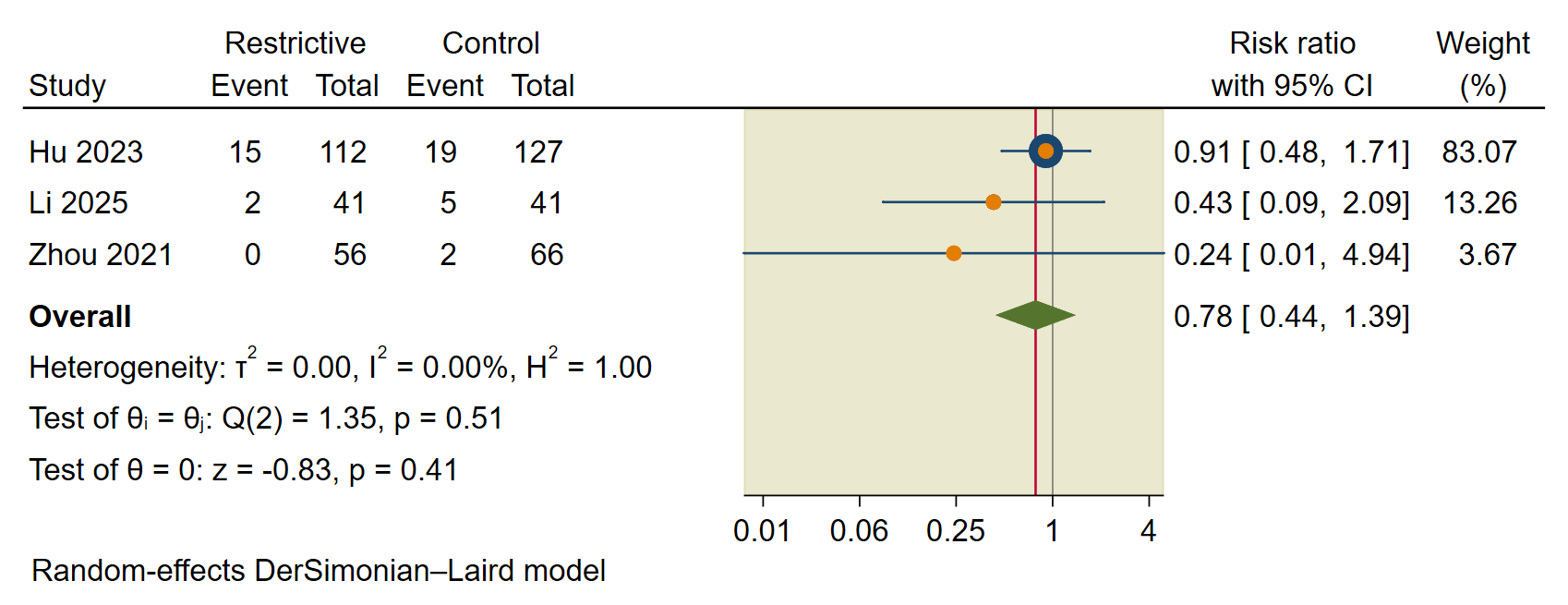

Supplement: Suppl 13 — Forest plot of multiple organ dysfunction syndrome (MODS). [file jocmr-18-03-177-s013.docx]

**Suppl 14.** Forest plot of intensive care unit (ICU) admission rate


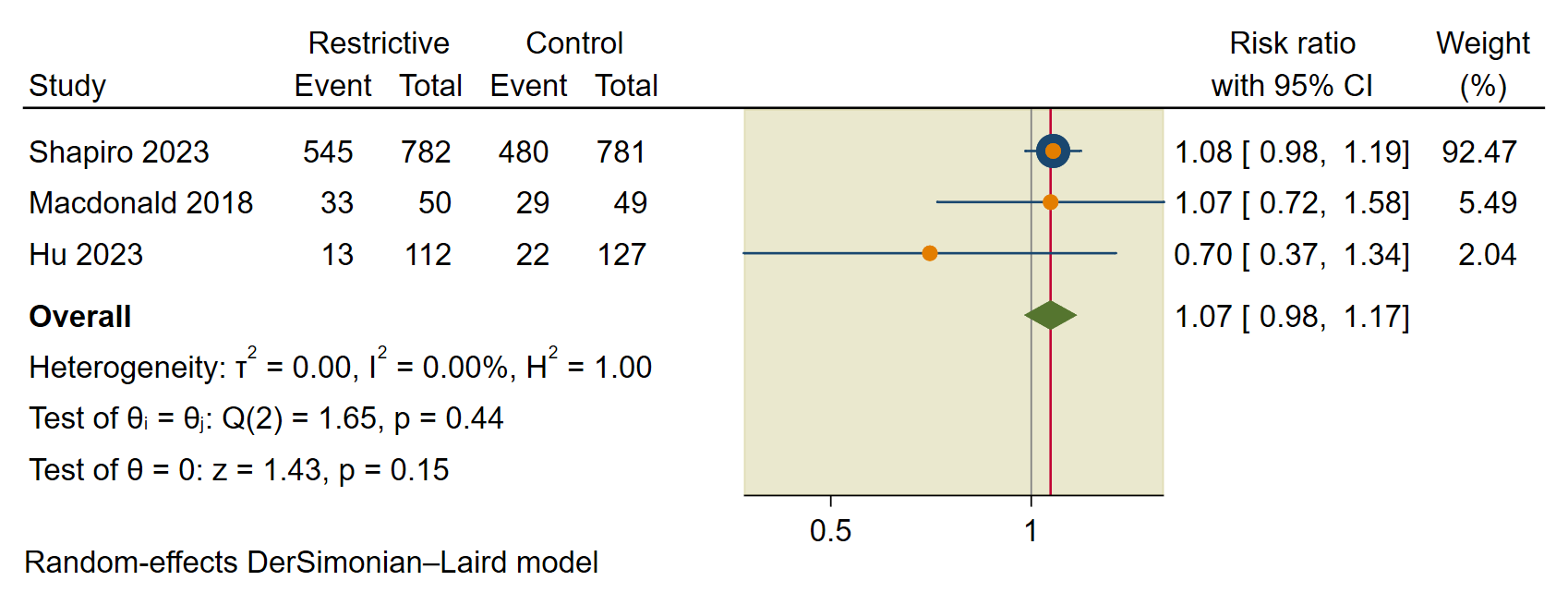

Supplement: Suppl 14 — Forest plot of intensive care unit (ICU) admission rate. [file jocmr-18-03-177-s014.docx]

**Suppl 15.** Forest plot of pulmonary edema


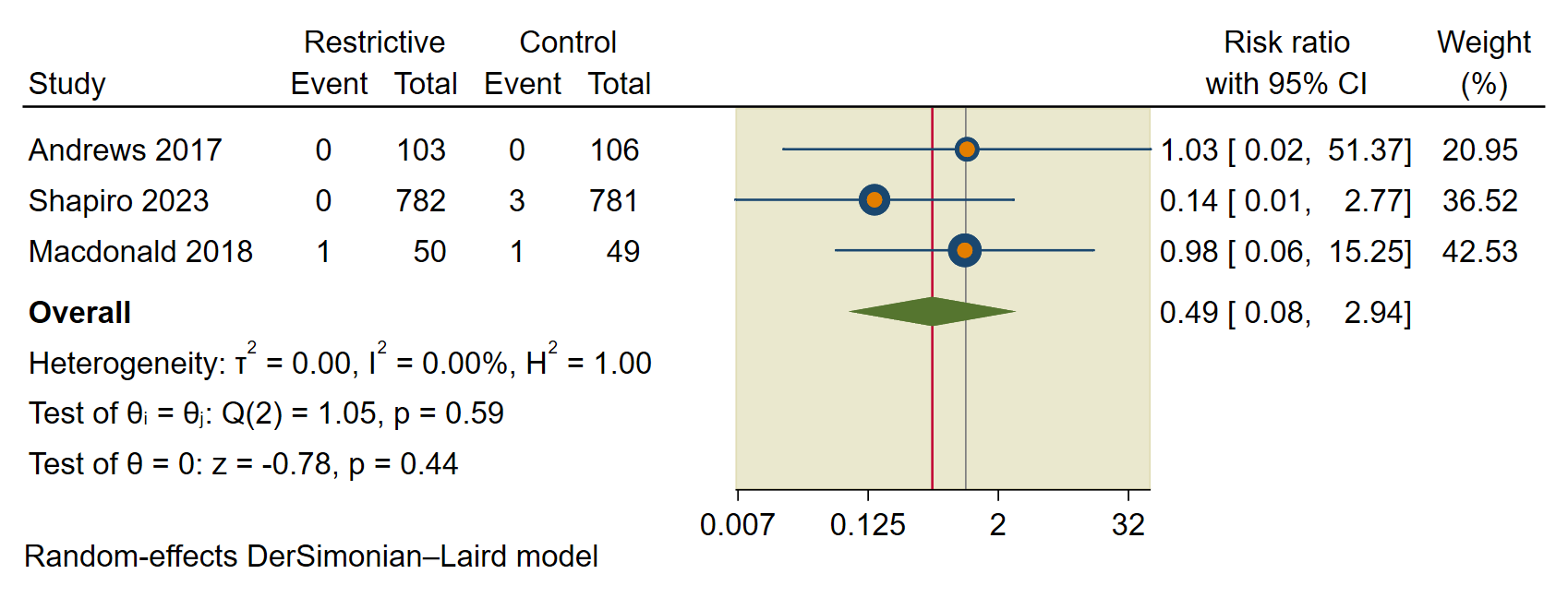

Supplement: Suppl 15 — Forest plot of pulmonary edema. [file jocmr-18-03-177-s015.docx]

**Suppl 16.** Forest plot of total serious adverse events


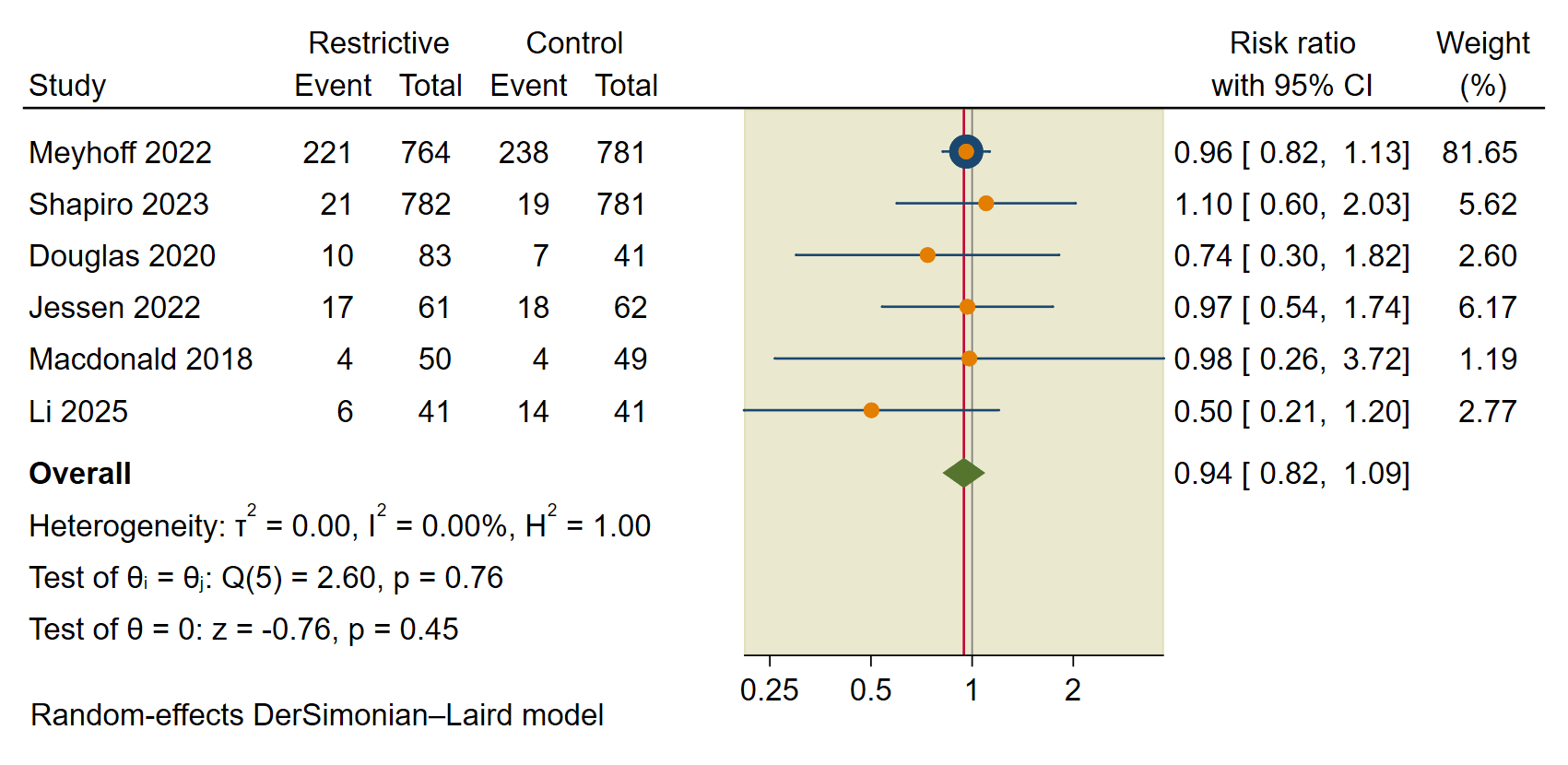

Supplement: Suppl 16 — Forest plot of total serious adverse events [file jocmr-18-03-177-s016.docx]

**Suppl 17.** Forest plot of incidence of acute respiratory distress syndrome


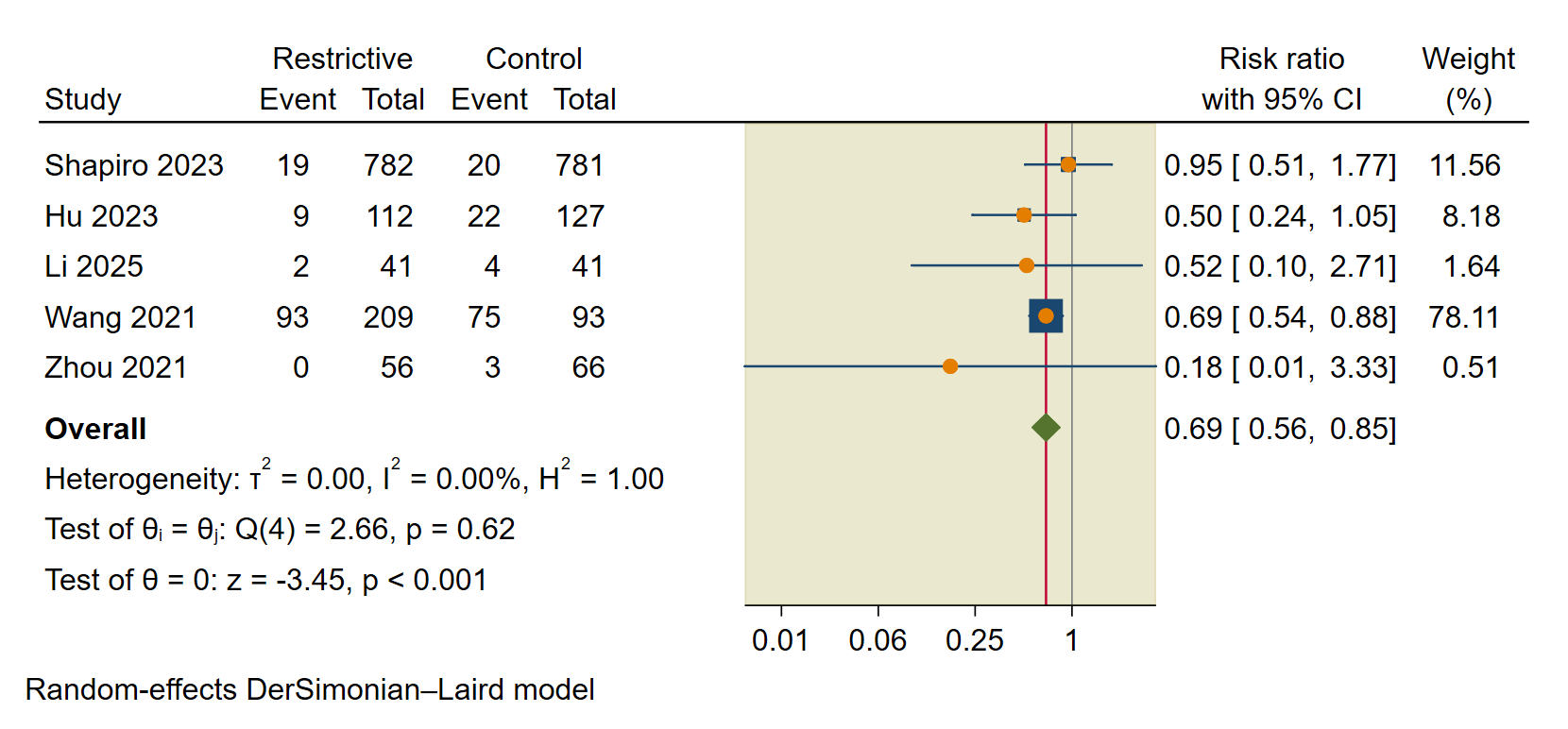

Supplement: Suppl 17 — Forest plot of incidence of acute respiratory distress syndrome. [file jocmr-18-03-177-s017.docx]

**Suppl 19.** Forest plot of hospital length of stay (days)


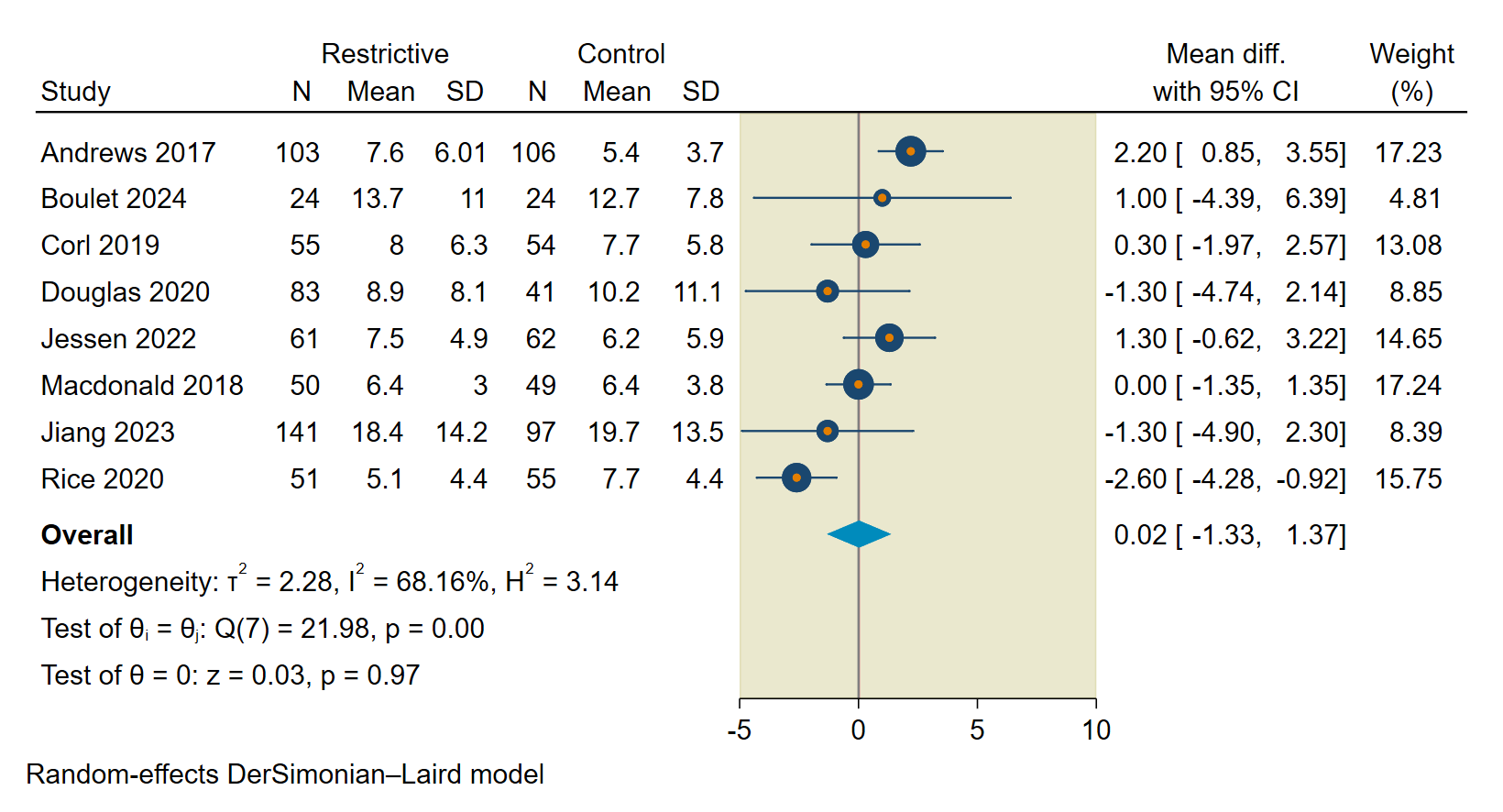

Supplement: Suppl 19 — Forest plot of hospital length of stay (days). [file jocmr-18-03-177-s019.docx]

**Suppl 20.** Forest plot of ICU length of stay (days)


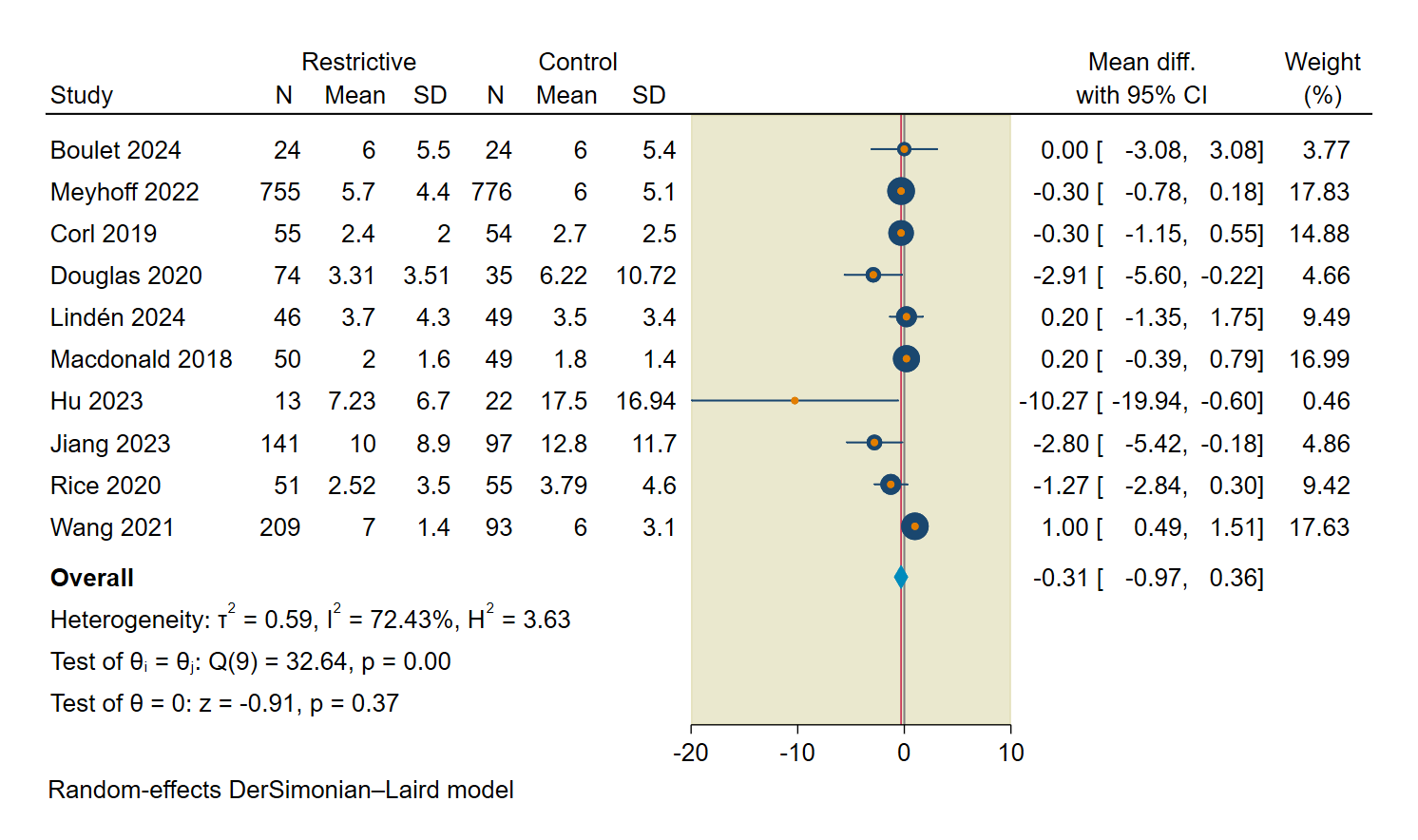

Supplement: Suppl 20 — Forest plot of ICU length of stay (days). [file jocmr-18-03-177-s020.docx]

**Suppl 21.** Forest plot of organ support-free days


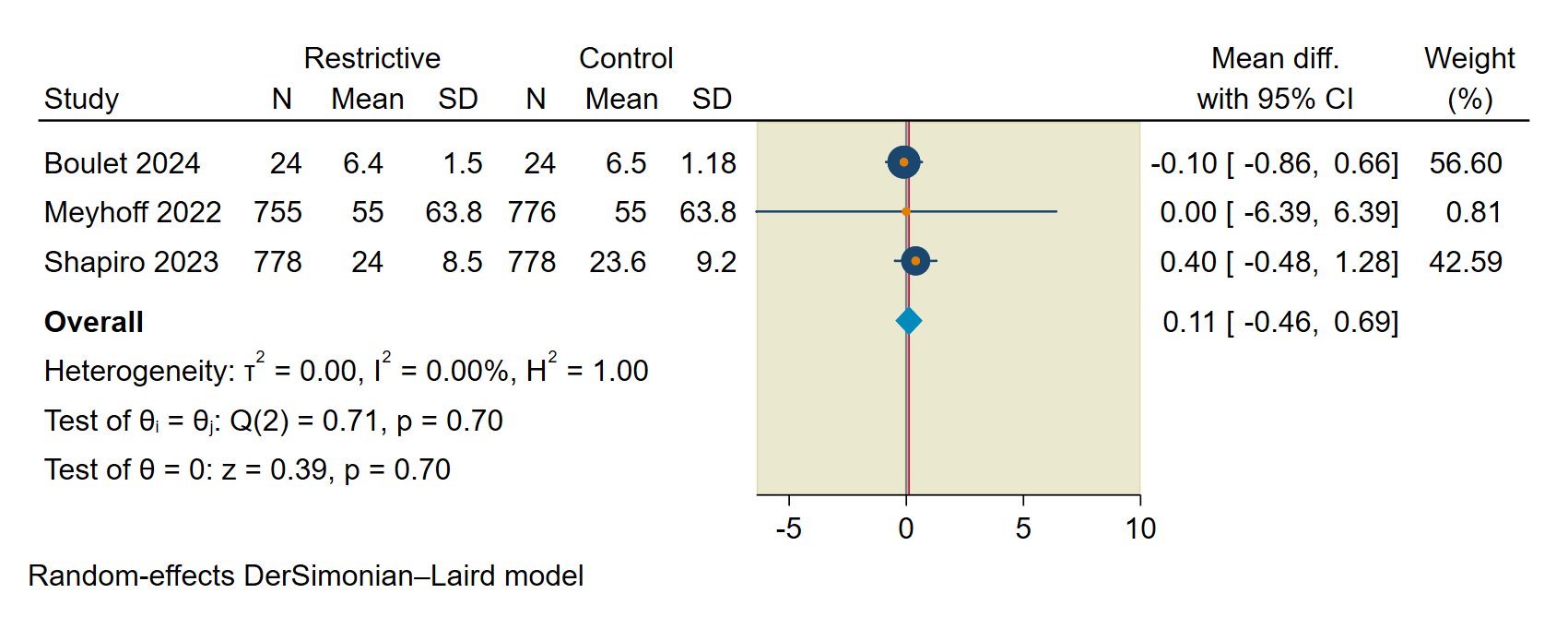

Supplement: Suppl 21 — Forest plot of organ support-free days. [file jocmr-18-03-177-s021.docx]

**Suppl 22.** Forest plot of vasopressor-free days


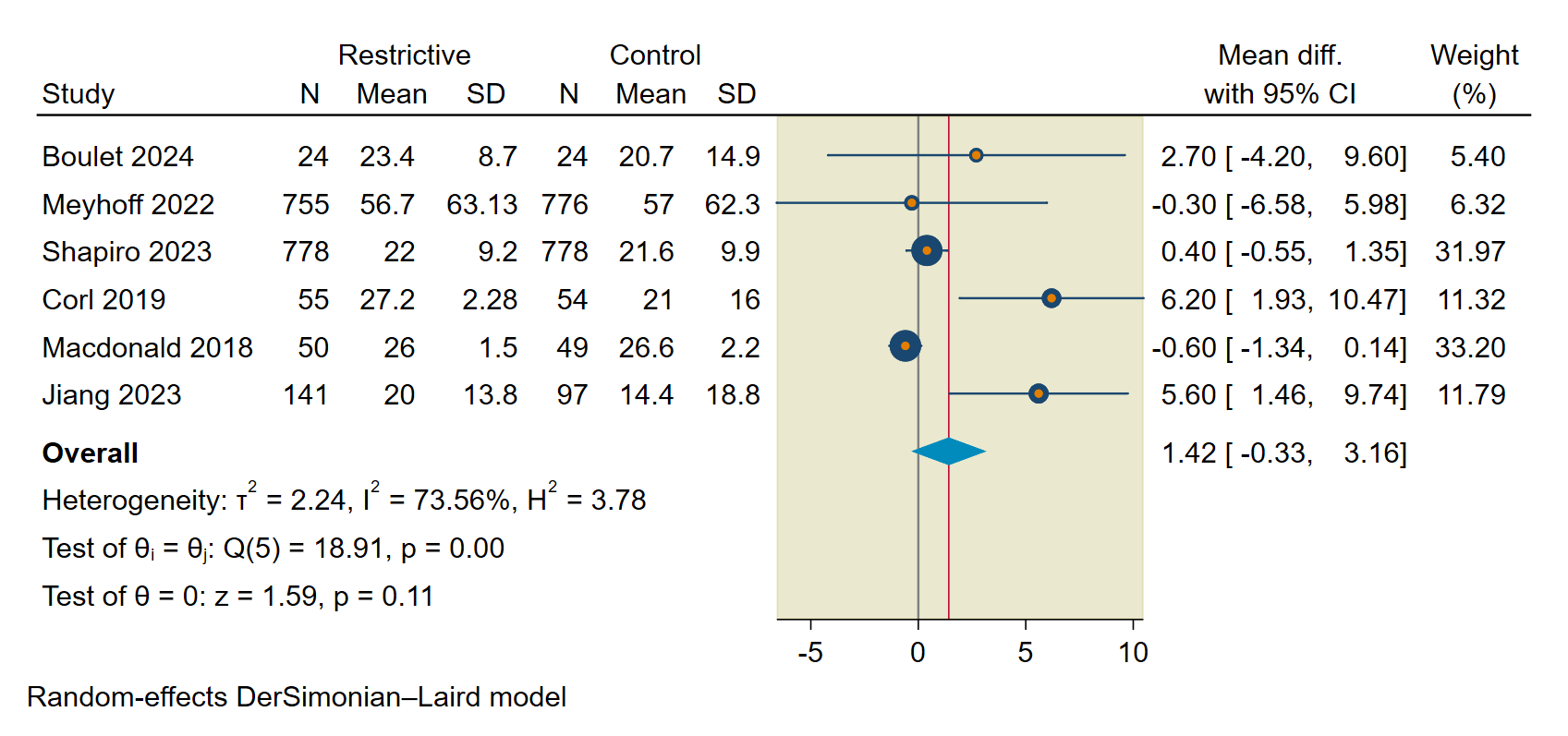

Supplement: Suppl 22 — Forest plot of vasopressor-free days. [file jocmr-18-03-177-s022.docx]

**Suppl 23.** Forest plot of vasopressin use


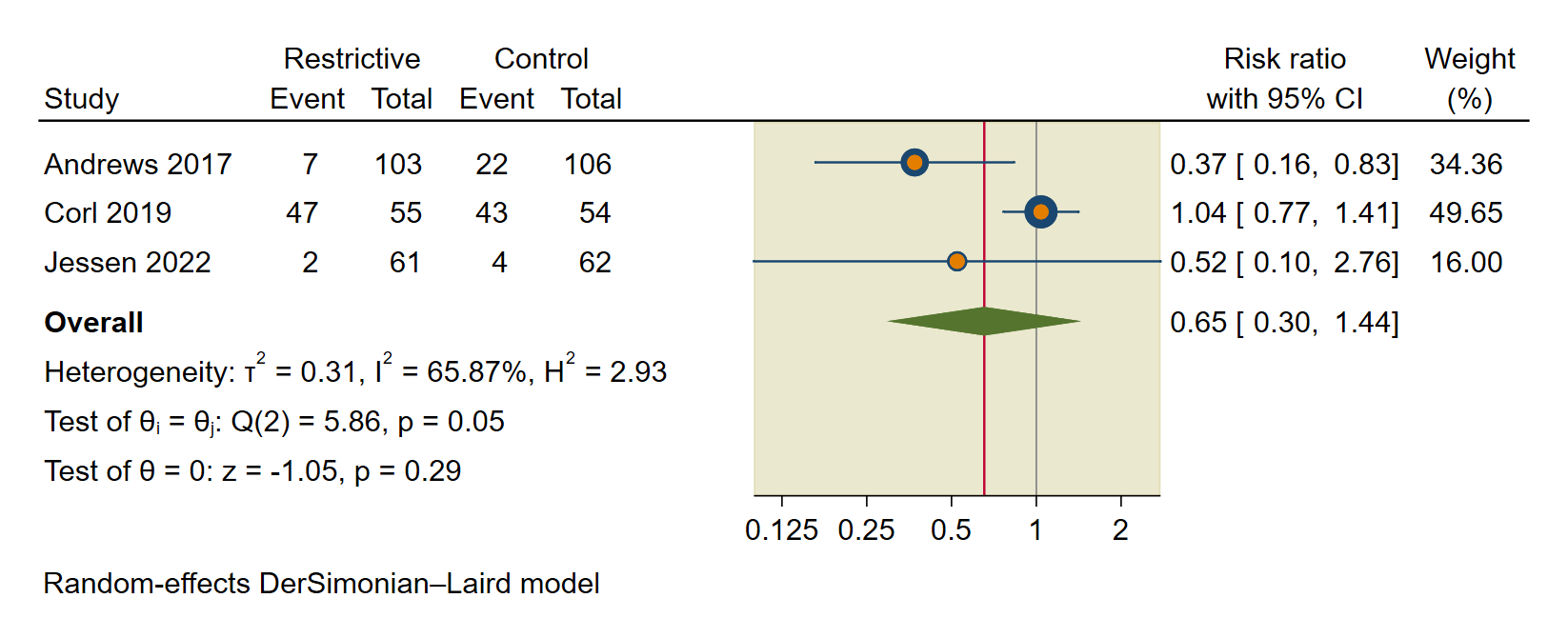

Supplement: Suppl 23 — Forest plot of vasopressin use. [file jocmr-18-03-177-s023.docx]

**Suppl 24.** Forest plot of initiation of renal replacement therapy


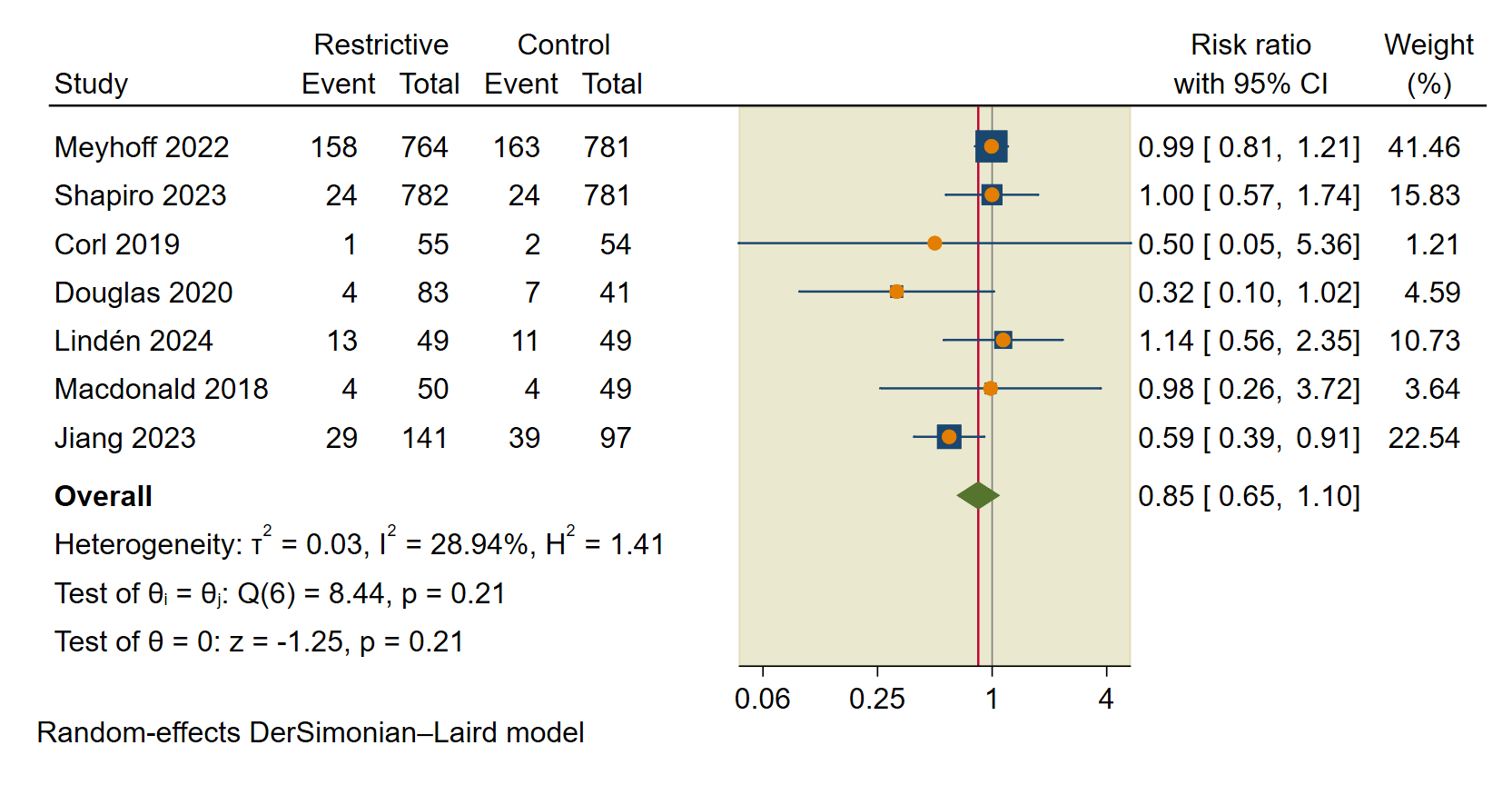

Supplement: Suppl 24 — Forest plot of initiation of renal replacement therapy. [file jocmr-18-03-177-s024.docx]

**Suppl 25.** Forest plot of the need for mechanical ventilation


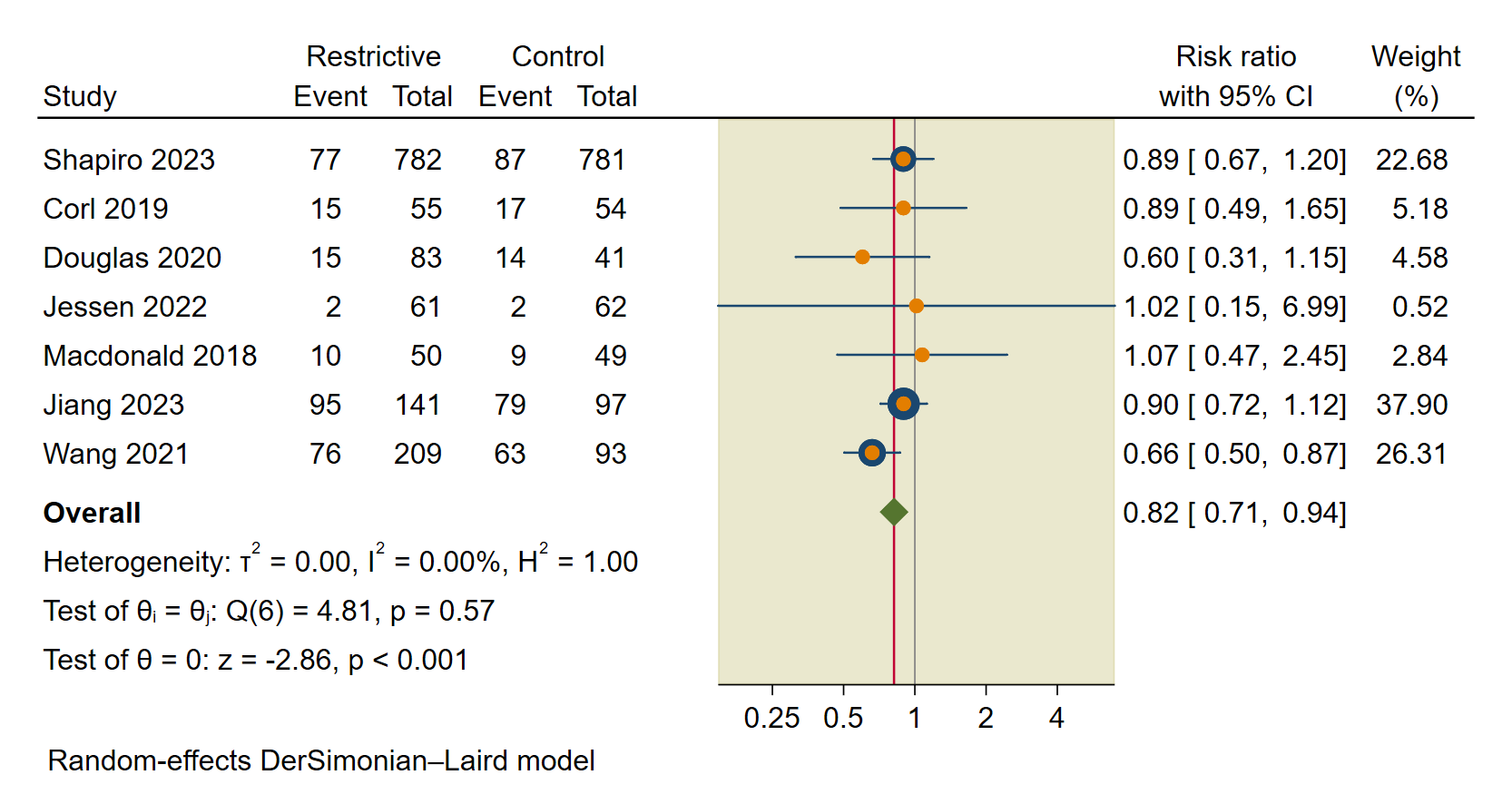

Supplement: Suppl 25 — Forest plot of the need for mechanical ventilation. [file jocmr-18-03-177-s025.docx]

**Suppl 26.** Forest plot of duration of mechanical ventilation (days)


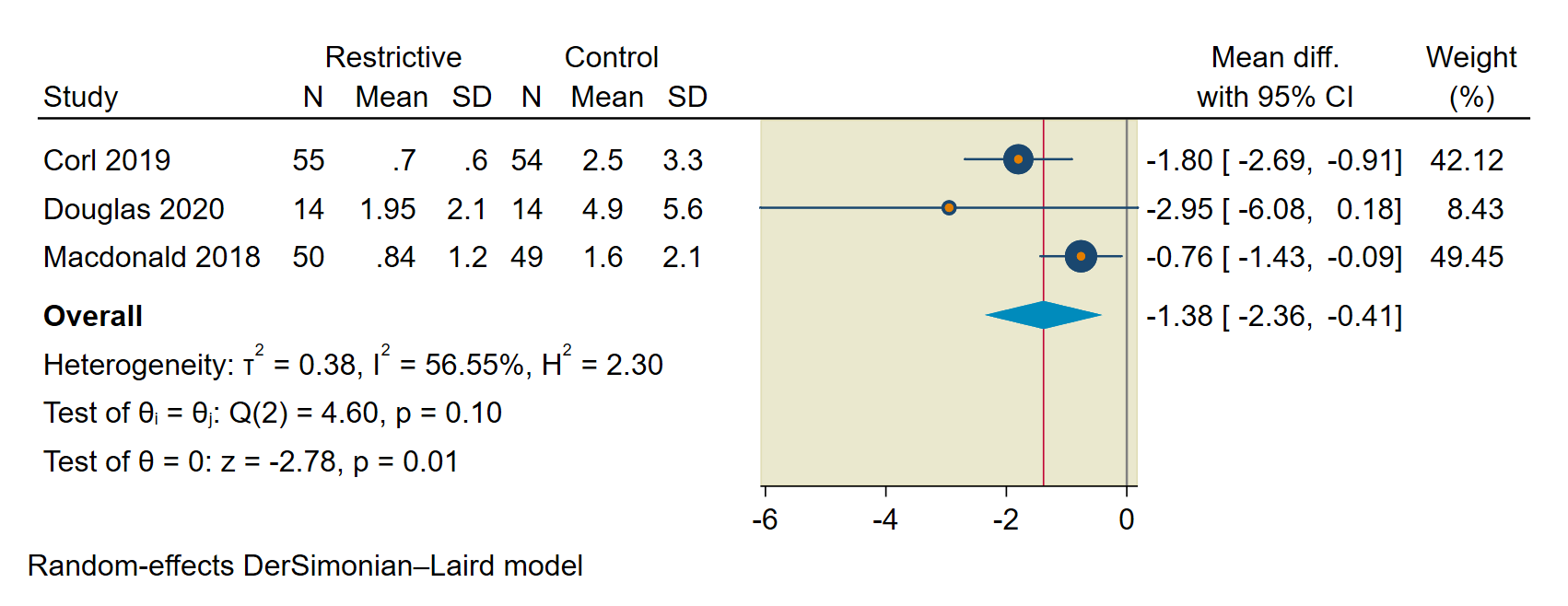

Supplement: Suppl 26 — Forest plot of duration of mechanical ventilation (days). [file jocmr-18-03-177-s026.docx]

**Suppl 27.** Forest plot of duration of vasopressin support (hours)


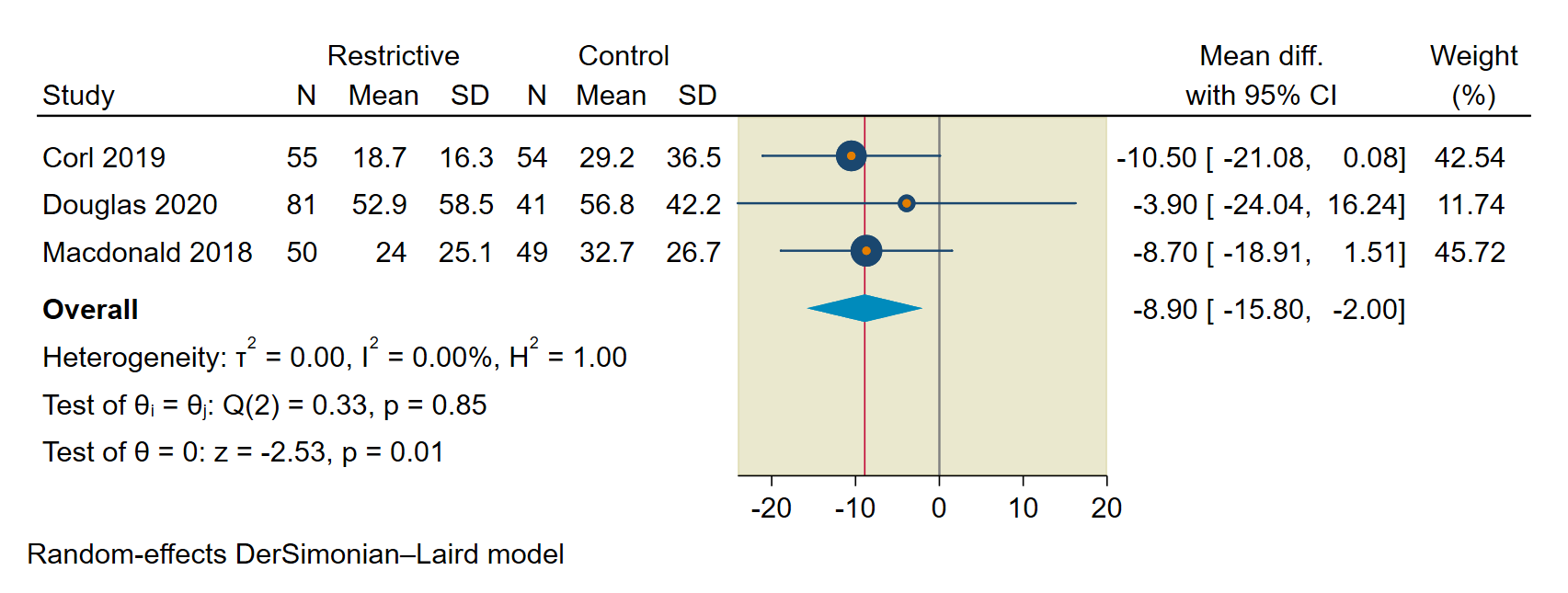

Supplement: Suppl 27 — Forest plot of duration of vasopressin support (hours). [file jocmr-18-03-177-s027.docx]

**Suppl 28.** Forest plot of ventilation-free days


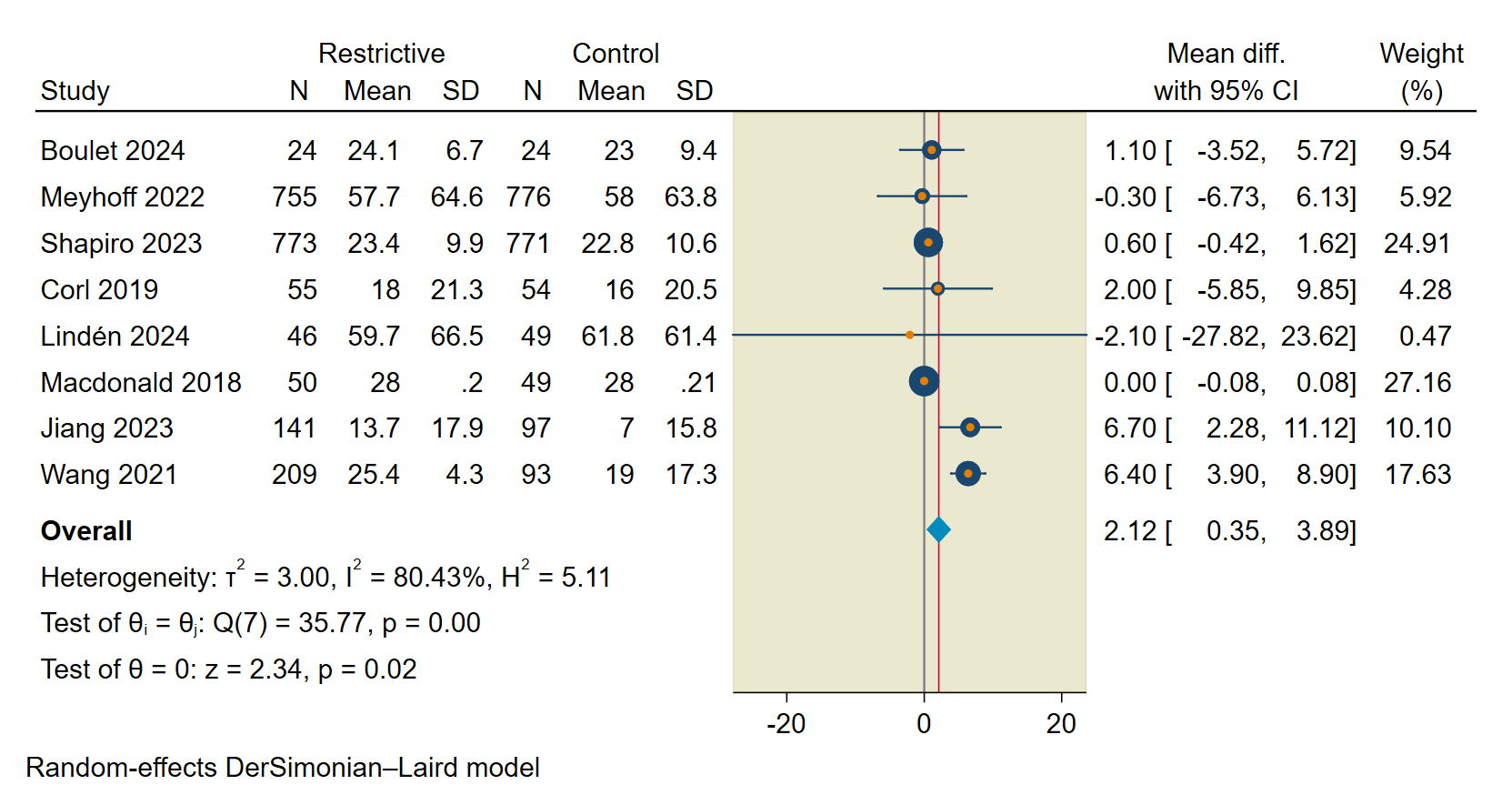

Supplement: Suppl 28 — Forest plot of ventilation-free days. [file jocmr-18-03-177-s028.docx]
