# Supplementary material for: Restrictive Versus Liberal Fluid Strategy for Initial Resuscitation in Sepsis and Septic Shock: A Systematic Review and Meta Analysis
Source: J Clin Med Res. 2026 Mar 26;18(3):177–95. doi: 10.14740/jocmr6464 (PMC13053473; doi:10.14740/jocmr6464)

**Suppl 11.** Forest plot of ischemic events, including cerebral ischemia, intestinal ischemia, limb ischemia, and myocardial ischemia


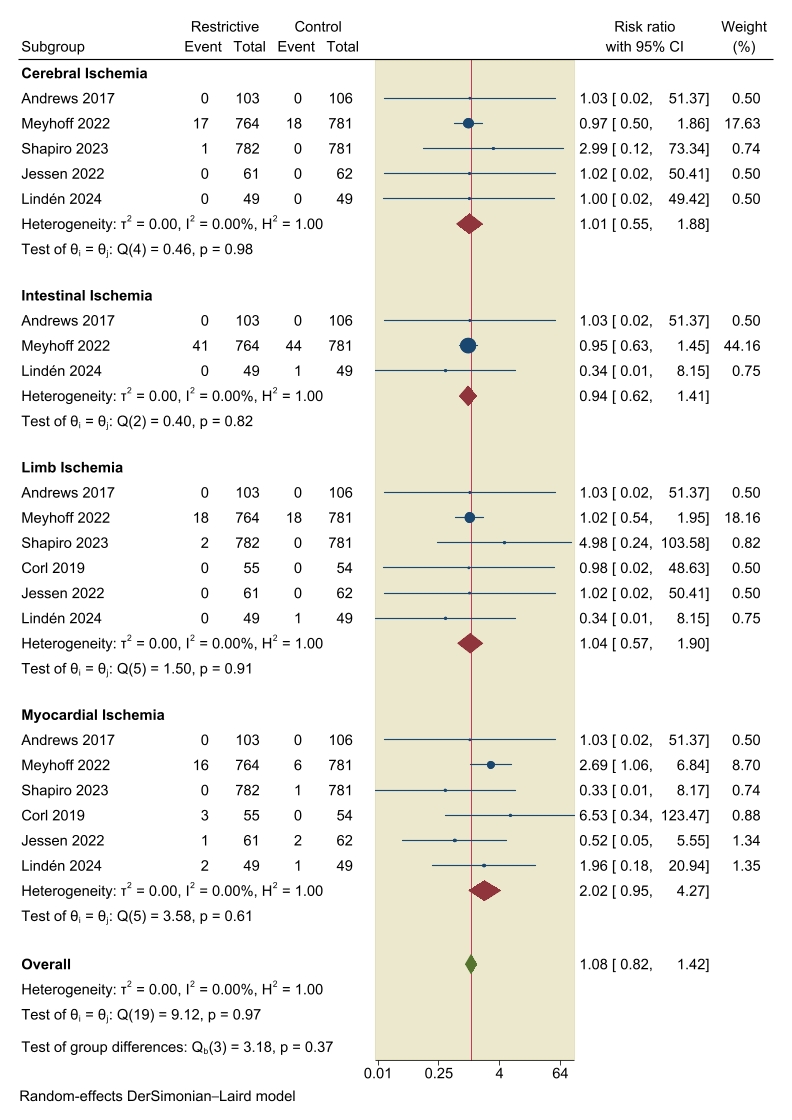

Supplement: Suppl 11 — Forest plot of ischemic events, including cerebral ischemia, intestinal ischemia, limb ischemia, and myocardial ischemia. [file jocmr-18-03-177-s011.docx]
