# Supplementary material for: Restrictive Versus Liberal Fluid Strategy for Initial Resuscitation in Sepsis and Septic Shock: A Systematic Review and Meta Analysis
Source: J Clin Med Res. 2026 Mar 26;18(3):177–95. doi: 10.14740/jocmr6464 (PMC13053473; doi:10.14740/jocmr6464)

**Suppl 18.** Forest plot of electrolyte and metabolic outcomes, including hyperchloremia, hypernatremia, hyponatremia, and hypoglycemia


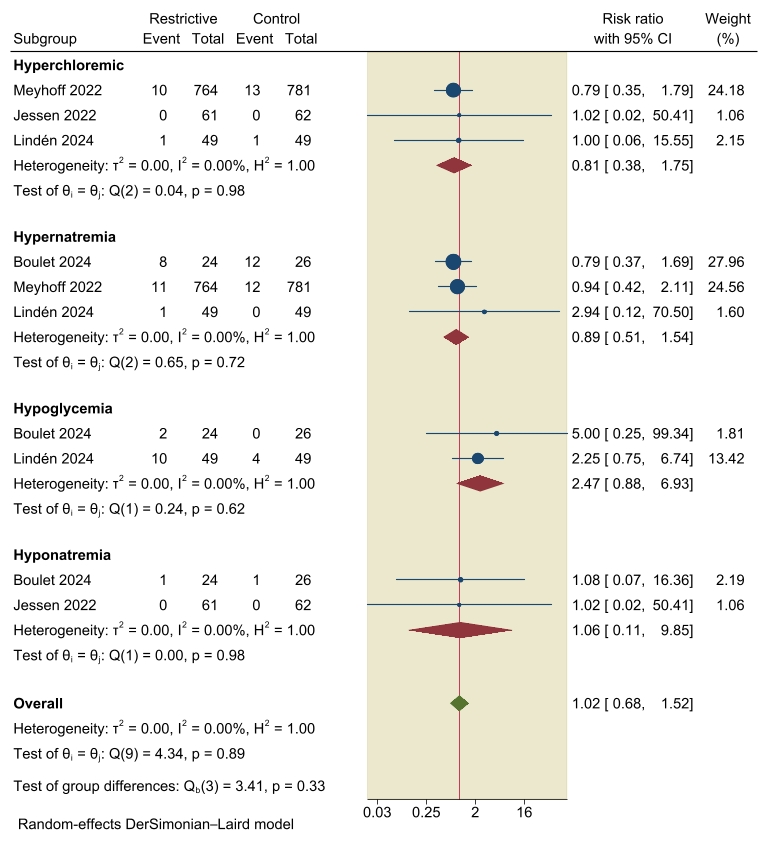

Supplement: Suppl 18 — Forest plot of electrolyte and metabolic outcomes, including hyperchloremia, hypernatremia, hyponatremia, and hypoglycemia. [file jocmr-18-03-177-s018.docx]
